# Metabolic reprogramming and epigenetic modifications on the path to cancer

**DOI:** 10.1007/s13238-021-00846-7

**Published:** 2021-05-29

**Authors:** Linchong Sun, Huafeng Zhang, Ping Gao

**Affiliations:** 1 Guangzhou First People’s Hospital, School of Medicine, Institutes for Life Sciences, South China University of Technology, Guangzhou, 510006 China; 2 The First Affiliated Hospital of USTC, University of Science and Technology of China, Hefei, 230027 China; 3 CAS Centre for Excellence in Cell and Molecular Biology, the CAS Key Laboratory of Innate Immunity and Chronic Disease, School of Basic Medical Sciences, Division of Life Sciences and Medicine, University of Science and Technology of China, Hefei, 230027 China; 4 School of Biomedical Sciences and Engineering, Guangzhou International Campus, South China University of Technology, Guangzhou, 510006 China; 5 Guangzhou Regenerative Medicine and Health Guangdong Laboratory, Guangzhou, 510005 China

**Keywords:** metabolic reprogramming, epigenetics, tumorigenesis, tumor immunity, cancer therapy

## Abstract

Metabolic rewiring and epigenetic remodeling, which are closely linked and reciprocally regulate each other, are among the well-known cancer hallmarks. Recent evidence suggests that many metabolites serve as substrates or cofactors of chromatin-modifying enzymes as a consequence of the translocation or spatial regionalization of enzymes or metabolites. Various metabolic alterations and epigenetic modifications also reportedly drive immune escape or impede immunosurveillance within certain contexts, playing important roles in tumor progression. In this review, we focus on how metabolic reprogramming of tumor cells and immune cells reshapes epigenetic alterations, in particular the acetylation and methylation of histone proteins and DNA. We also discuss other eminent metabolic modifications such as, succinylation, hydroxybutyrylation, and lactylation, and update the current advances in metabolism- and epigenetic modification-based therapeutic prospects in cancer.

**Supplementary Information:**

The online version contains supplementary material available at (10.1007/s13238-021-00846-7) contains supplementary material, which is available to authorized users.

## PATHWAYS LEADING TO THE INTEGRATION OF METABOLISM AND EPIGENETIC MODIFICATION DURING CANCER DEVELOPMENT

**Figure 1:**
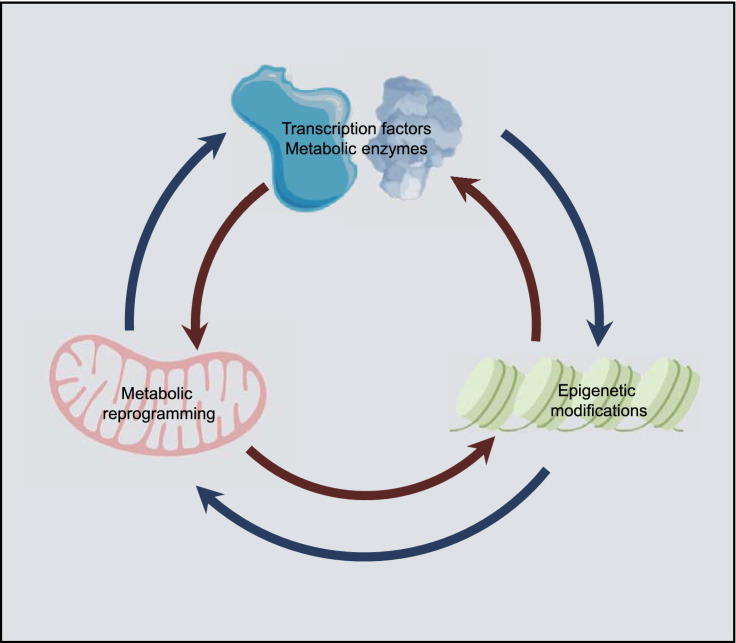
**Crosstalks between metabolic reprogramming, epigenetic modifications, and transcriptional regulation**. The cell metabolome and epigenome interact in a two-way manner and with genetic and molecular drivers that regulate cancer. A comprehensive understanding of the interactions between molecular drivers, metabolic reprogramming, and epigenetic modifications in cancer will further elucidate their connections and contribute to the development of effective cancer therapies

Metabolic reprogramming is one of the major features of cancer, during which characteristics of metabolic enzymes, upstream regulating molecules and downstream metabolic products, known as metabolites, are altered (DeBerardinis et al., 2008b; Heiden et al., 2009; Jones and Thompson, 2009; Hanahan and Weinberg, 2011; DeBerardinis and Thompson, 2012; Hirschey et al., 2015; DeBerardinis and Chandel, 2016; Pavlova and Thompson, 2016; Sun et al., 2018; Thompson, 2019; Dai et al., 2020; Faubert et al., 2020). Recently, metabolism has been regarded as a major player and context-dependent regulator of epigenetic modifications, and increasing evidence suggests that intermediary metabolites drive chromatin dynamics through chemical posttranslational modifications (PTMs) that alter chromatin structures and functions (Kaelin and McKnight, 2013; Janke et al., 2015; Keating and El-Osta, 2015; Parker and Metallo, 2016; Reid et al., 2017; Chisolm and Weinmann, 2018; Wang and Lei, 2018; Zheng et al., 2020). Cellular metabolism and the epigenome interact in a bidirectional manner and interact with the genetic and molecular drivers that regulate cancer (Fig. 1). However, a comprehensive understanding of the interactions between molecular drivers, metabolic reprogramming, and epigenetic modifications in cancer are lacking, and thus, further elucidation of the associations is both necessary and pressing for more effective cancer therapy.

**Table 1:** Acetylation regulates the subcellular locations, activity, and corresponding biological functions of transcription factors and metabolic enzymes

Proteins	Acetylation sites	Acetyltransferase	Deacetylase	Location	Function	Roles in ancer	References
c-Myc	Mouse (and human) c-Myc: K144 (143), K149 (148), K158 (157), K275, K317, K323, and K371	p300	HADC	N/A in this paper	Dual roles for p300/CBP in Myc regulation: as a Myc coactivator that stabilizes Myc and as an inducer of Myc instability via direct Myc acetylation	N/A in this paper	Faiola et al., 2005
HIF-1α	K674	PCAF	SIRT1	N/A in this paper	Acetylation increases HIF-1α activity	SIRT1 has negative effects on tumor growth and angiogenesis by deacetylation of HIF-1α	Lim et al., 2010
GAPDH	K254	PCAF	HDAC5	N/A in this paper	K254 acetylation increases GAPDH enzyme activity	Acetylation of GAPDH at K254 promotes tumor cell proliferation and tumor growth in xenograft model	Li et al. 2014
GAPDH	K117, K251	PCAF	N/A in this paper	Nuclear translocation	PCAF-mediated GAPDH acetylation induces the nuclear translocation of GAPDH	N/A in this paper	Ventura et al., 2010
PKM2	K433	p300	N/A in this paper	Nuclear translocation	Acetylation prevents PKM2 activation by interfering with FBP binding and promotes the nuclear accumulation and protein kinase activity of PKM2	Acetylation-mimetic PKM2 (K433) mutant promotes cell proliferation and tumorigenesis	Lv et al., 2013
PEPCK	K70, K71, K594	p300	SIRT2	N/A in this paper	Acetylation promotes ubiquitin-proteosome degradation of PEPCK1 and decreases its activity	N/A in this paper	Jiang et al., 2011
6PGD	K76, K294	DLAT, ACAT2	HDAC4	N/A in this paper	Acetylation at K76 and K294 promotes NADP^+^ binding to 6PGD and actives 6PGD dimer formation	Acetylation of 6PGD K76 and K294 is important for cancer cell proliferation and tumor growth	Shan et al., 2014
ACLY	K540, K546, K554	PCAF	SIRT2	N/A in this paper	Acetylation stabilizes ACLY by inhibiting ubiquitylation and ACLY acetylation promotes *de novo* lipid synthesis	Acetylation of ACLY promotes lipogenesis and tumor cell proliferation and is increased in lung cancer	Lin et al., 2013
LCAD	K42	N/A in this paper	SIRT3	N/A in this paper	Hyperacetylation of LCAD reduces its enzyme activity	N/A in this paper	Hirschey et al., 2010
LCAD	K318, K322	N/A in this paper	SIRT3	N/A in this paper	Acetylation/deacetylation at Lys-318/Lys-322 is a mode of regulating fatty acid oxidation	N/A in this paper	Bharathi et al., 2013
BCAT2	K44	CBP	SIRT4	N/A in this paper	Acetylation promotes BCAT2 degradation via ubiquitylation without affecting its enzyme activity	BCAT K44R mutant promotes BCAA catabolism, cell proliferation, and pancreatic tumor growth	Lei et al., 2020

N/A, not applicable.

Cellular chromatin is composed of DNA and histones. Histones can undergo a wide range of PTMs such as phosphorylation, methylation, acetylation, and other acylation modifications. Similar to histones, DNA and RNA can be chemically modified by methylation to regulate gene expression. Epigenetic characteristics are usually abnormal in cancer cells. Human cancers often exhibit characteristic changes in DNA methylation, including genome-wide hypomethylation and site-specific hypermethylation (Jones and Baylin, 2002; Feinberg and Tycko, 2004). Global DNA hypomethylation in cancer was first observed by the Bert Vogelstein group in 1983 (Feinberg and Vogelstein, 1983). In mice, DNA hypomethylation is sufficient to induce aggressive T-cell lymphomas with a high frequency of chromosome 15 trisomy (Eden et al., 2003; Gaudet et al., 2003), whereas tumor suppressor genes are usually silenced by site-specific DNA hypermethylation at their promoters (Esteller et al., 2001). Similarly, the loss of histone 4 lysine 16 acetylation or histone 4 lysine 20 trimethylation is a common hallmark of human cancers (Fraga et al., 2005). Low levels of histone 3 lysine 4 dimethylation are associated with poor prognosis for patients with prostate (Seligson et al., 2005; Bianco-Miotto et al., 2010), lung (Barlesi et al., 2007; Seligson et al., 2009), breast (Elsheikh et al., 2009), pancreas (Manuyakorn et al., 2010), or kidney cancer (Ellinger et al., 2010). In addition, many oncogenes and tumor suppressors such as hypoxia-inducible factors (HIFs) (Watson et al., 2010; Prickaerts et al., 2016; Nanduri et al., 2017), von Hippel-Lindau tumor suppressor (VHL) (Herman et al., 1994; Schmitt et al., 2009; Vanharanta et al., 2013), Myc (Dang, 2012; Stine et al., 2015; Poole and van Riggelen, 2017; Topper et al., 2017; Poli et al., 2018; Li et al., 2020), p53 (Vrba et al., 2008; Su et al., 2009; Saldana-Meyer and Recillas-Targa, 2011), phosphatase and tensinhomolog (PTEN) (Salvesen et al., 2001; Kang et al., 2002; Soria et al., 2002; Garcia et al., 2004; Alvarez-Nunez et al., 2006), liver kinase B1 (LKB1) (Esteller et al., 2000; Trojan et al., 2000), AMP-activated protein kinase (AMPK) (Ruderman et al., 2010; Gongol et al., 2018; Yuan et al., 2020), and mechanistic target of rapamycin kinase (mTOR) (Laribee, 2018; Zeng et al., 2019), drive epigenetic reprogramming and are regulated by epigenetic modifications (Fig. 1).

Epigenetic abnormalities regulate the expression of many metabolic genes, thus playing important roles in metabolic rewiring and redox homeostasis of cancer cells (Wong et al., 2017). In contrast, metabolic flux is involved in epigenetic regulation by affecting the biosynthesis of macromolecules and energy production (Zheng et al., 2020). All these events are synergistically involved in the path to cancer. For example, in addition to regulating glucose, glutamine and serine metabolism at the transcriptional level (Gao et al., 2009; Stine et al., 2015; Sun et al., 2015; Wu et al., 2017), cMyc increases SDHA (succinate dehydrogenase complex, subunit A) acetylation by promoting SKP2 (S-phase kinase-associated protein 2)-mediated sirtuin3 degradation, leading to SDHA deactivation and succinate accumulation. Increased succinate inhibits the activity of histone demethylases, which triggers histone 3 lysine 4 trimethylation and the expression of tumor-specific genes and subsequent tumor progression (Li et al., 2020). During pancreatic ductal adenocarcinoma (PDAC) progression, 6-phosphogluconate dehydrogenase (6PGD) -mediated oxidative pentose phosphate pathway (oxPPP) supports the reprogramming of histone H3K9 and DNA methylation, thereby promoting N-cadherin (epithelial-mesenchymal transition marker) transcription and N-cadherin-mediated distant metastasis (McDonald et al., 2017). SETD2 (SET domain-containing 2, a histone lysine methyltransferase) integrates EZH2 (enhancer of zeste homolog 2) and the AMPK signaling pathway to restrict prostate cancer metastasis by linking metabolism with epigenetic modifications (Yuan et al., 2020). H3.3K27M (histone H3.3 lysine 27-to-methionine) mutation in diffuse intrinsic pontine gliomas (DIPGs) results in global H3K27me3 reduction by multiple mechanisms, such as the aberrant PRC2 interactions or hampered H3K27me3 spreading (Bender et al., 2013; Chan et al., 2013; Lewis et al., 2013; Stafford et al., 2018; Harutyunyan et al., 2019). However, by integrating metabolic and epigenetic pathways, Chung et al. found that H3.3K27M mutations promote glycolysis, glutaminolysis, and TCA cycle-derived α-KG (α-ketoglutarate) accumulation, leading to α-KG-dependent activation of H3K27 demethylases KDM6A/6B, H3K27 hypomethylation, and tumor progression (Chung et al., 2020; Zhao and Miao, 2020). Histone acetylation regulates cell proliferation and tumor progression (Cai et al., 2011; Donohoe et al., 2012; Lee et al., 2014), as well as other cellular biological behaviors not covered in this review article, such as intracellular pH (McBrian et al., 2013), hippocampal memory (Mews et al., 2017), cell fate decisions (Yadav et al., 2018), and cellular differentiation (Chisolm and Weinmann, 2018).

**Figure 2:**
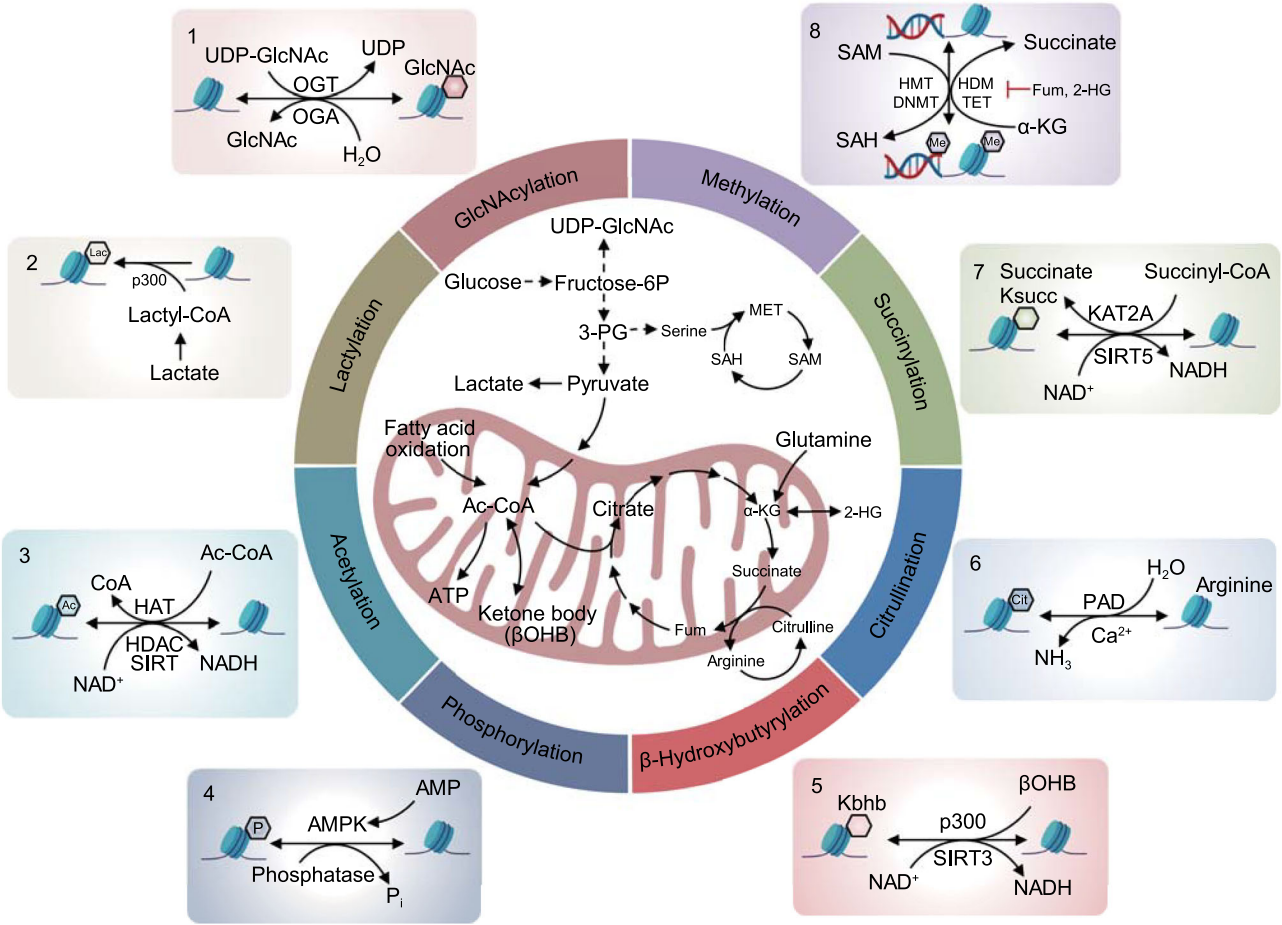
**An overview of metabolic connections to epigenetic remodeling**. Nutrients such as glucose, fatty acids, and amino acids are metabolized by cells to produce a variety of metabolites, such as acetyl-CoA, NAD^+^, SAM, α-KG, ATP, and succinate, which function as substrates or cofactors to modify chromatin and proteins. Specifically, 1) UDP-GlcNAc, as a donor substrate derived from the HBP pathway integrating glucose, glutamine, fatty acid (acetyl-CoA), and nucleotide metabolism (UDP), is catalyzed by OGT for GlcNAcylation modification, and OGA controls the reverse reaction. 2) Lactate generates lactyl-CoA, which contributes a lactyl group to lysine residues of histone proteins through p300, generating a novel modification called lactylation. 3) Glucose-, fatty acid-, amino acid-, and acetate-derived acetyl-CoA are widely involved in acetylation modification. Histone acetylation is catalyzed by HATs, and the reverse reaction is mediated by lysine deacetylases (HDAC and SIRT). 4) Based on the ratio of ATP:AMP, AMPK is required for the phosphorylation of histones under various stress conditions. 5) Histone lysine β-hydroxybutyrylation (Kbhb) depends on the metabolite β-hydroxybutyrate (βOHB), which is produced by the ketone body metabolic pathway. The enzymes involved in acetylation modification mediate this reversible reaction. 6) Citrulline is categorized into two types: free citrulline from the arginine-coupled urea cycle and the guanidine dehydration of arginine residues on proteins to create citrulline residues. Histone citrullination is a PTM that converts arginine residues to citrulline via PAD enzymes, which are Ca^2+^-dependent. 7) TCA cycle-derived succinyl-CoA is the major substrate for succinylation, and the opposite reaction is mediated by KAT2A, CPT1A, and SIRT5. 8) Reversible chromatin methylation is coupled with SSP, the folate cycle, and the methionine cycle. SAM is the substrate for HMTs and DNMTs, leading to the production of SAH. Succinate, fumarate, and 2-HG inhibit the demethylases HDMs and TETs, which catalyze the demethylation reaction in an α-KG-dependent manner. In addition, NAD^+^ and NADH transitions are involved in modifications such as acetylation, β-hydroxybutyrylation, and succinylation

Notably, all these events and reactions require metabolites, including acetyl-CoA, NAD^+^ (nicotinamide adenine dinucleotide), SAM (S-adenosyl methionine), α-KG, FAD (flavin adenine dinucleotide), ATP, and succinate, as substrates or cofactors (Fig. 2). The dysregulation of histone PTMs and DNA/RNA modifications is associated with the occurrence of many diseases. Although all these metabolites play crucial roles in energy metabolism, cell cycle progression, cell growth and death, neuroregeneration, circadian rhythm, and the pluripotency of stem cells, in this review, we discuss the current understanding of how essential metabolites, as well as their regulating molecules, control the epigenome by dynamically regulating the metabolic states of DNA, histones and other proteins during cancer development.

## METABOLITES PLAY KEY ROLES IN EPIGENETIC REMODELING ON THE PATH TO CANCER

### Acetyl-CoA metabolism in acetylation regulation

#### Writers, readers, and erasers of protein acetylation

Protein (histone) acetylation is a chemical reaction catalyzed by lysine (histone) acetyltransferases (KATs/HATs), during which an acetyl group donated by acetyl-CoA is added to a lysine residue of the protein (histone). Three major families of KATs, GNAT (G protein subunit alpha transducin), MYST (Moz, Ybf2/Sas3, Sas2, and Tip60), and p300/CBP (E1A-binding protein p300/CREB-binding protein), have been identified (Sabari et al., 2017). All these KATs require acetyl-CoA, the sole donor of the acetyl group in eukaryotic cells (Choudhary et al., 2014). Bromodomain proteins (e.g., BRD4 and BRDT), YEATS domain proteins (e.g., MLLT3 and Taf14), and double PHD finger (DPF) domain proteins (e.g., MOZ and DPF2) are readers that interact with acetyl-lysine residues and recognize the lysine acetylation (K_acetyl_) (Sabari et al., 2017) to recruit transcription factors and/or super elongation complexes to support transcriptional activation (Fujisawa and Filippakopoulos, 2017; Gates et al., 2017; Zhao et al., 2017; Haws et al., 2020). Lysine deacetylases are erasers critical for removing acetyl groups. Zinc-dependent histone deacetylases (zinc-dependent HDACs) and NAD^+^-dependent sirtuins are two major families of lysine deacetylases (De Ruijter et al., 2003; Jing and Lin, 2015). Class I (HDAC1, 2, 3, and 8), class II (HDAC4, 5, 6, 7, 9, and 10), and class IV (HDAC11) HDACs are zinc-dependent enzymes, and class III HDACs, also called sirtuins, are dependent on the NAD^+^ concentration (Fig. 2).

In most mammalian cells, acetyl-CoA is a central metabolite that is primarily generated from glucose-derived pyruvate by the pyruvate dehydrogenase complex (PDC) in mitochondria. Fatty acid β-oxidation (Rufer et al., 2009), the catabolism of branched amino acids (BCAAs) (Harris et al., 2005), and free acetate all contribute to the generation of mitochondrial acetyl-CoA (Pietrocola et al., 2015) (Fig. 2); however, there is no acetyl-CoA transporter on the mitochondrial membrane. In rapidly proliferating cells, citrate, upon synthesis due to acetyl-CoA and oxaloacetate (OAA) condensation in mitochondria, is quickly exported to the cytosol by the citrate carrier SLC25A1, where it is converted back to acetyl-CoA and OAA by ATP citrate lyase (ACLY) (Icard et al., 2012; Zaidi et al., 2012). Both ACLY and all the subunits of PDC are present in the nucleus of mammalian cells and promote the generation of acetyl-CoA (Wellen et al., 2009; Sutendra et al., 2014). Acetyl-CoA synthesis from acetate is mediated by acyl-CoA synthetase short-chain family members (ACSSs), including ACSS1 and ACSS3 in mitochondria and ACSS2 in the cytoplasm and nucleus (Luong et al., 2000; Fujino et al., 2001; Perez-Chacon et al., 2009; Ariyannur et al., 2010; Choudhary et al., 2014; Comerford et al., 2014). Acetyl-CoA functions as a carbon source for histone acetylation, cell growth and proliferation (Cai et al., 2011) and regulates autophagy (Eisenberg et al., 2014) and intracellular pH (McBrian et al., 2013). Here, we focus on localized acetyl-CoA production mediated by PDC, ACLY, and ACSSs in different organelles and its regulation of chromatin and other proteins.

#### The roles of compartmentalized acetyl-CoA metabolism in chromatin regulation and protein acetylation

*PDC* Glucose-derived cytosolic pyruvate enters mitochondria by the mitochondrial pyruvate carrier (MPC), a heterodimer of MPC1 and MPC2 (Herzig et al., 2012). Mitochondrial pyruvate is decarboxylated to generate acetyl-CoA by PDC, a large multicomponent composed of pyruvate dehydrogenase (PDH), dihydrolipoamide S-acetyltransferase (DLAT), dihydrolipoamide dehydrogenase (DLD), pyruvate dehydrogenase kinase (PDK), pyruvate dehydrogenase phosphatase (PDP), and pyruvate dehydrogenase complex, component X (PDHX). Among these proteins, PDH, DLAT, and DLD are directly involved in CoA- and NAD^+^-dependent pyruvate decarboxylation; PDK and PDP are two regulatory components; and PDHX is a nonenzymatic subunit (Patel et al., 2014).

**Figure 3:**
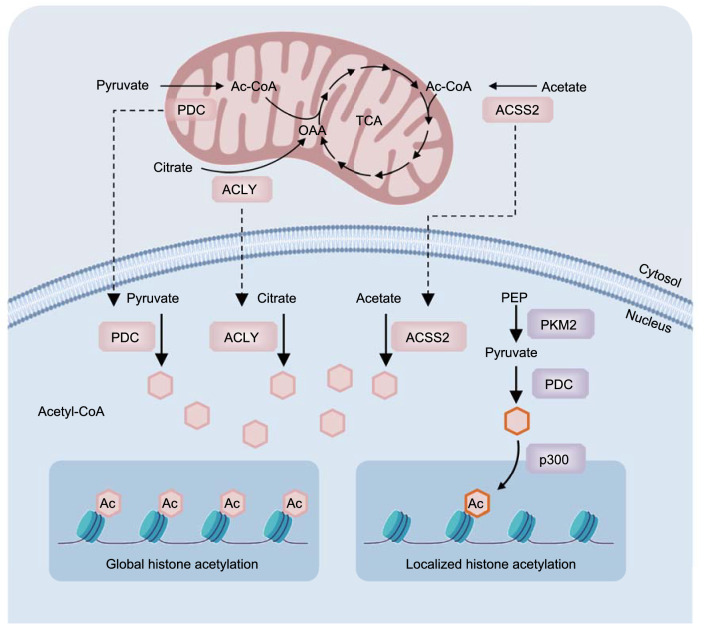
**Compartmentalized acetyl-CoA metabolism in chromatin regulation**. Under stimulation or stress conditions, mitochondrial-localized PDC and cytosol-localized ACLY and ACSS2 may translocate into the nucleus for the generation of the nuclear acetyl-CoA pool, mediating global histone acetylation (left). In certain cases, PDC binds with PKM2 and p300 to generate a large complex in the nucleus. In this large nuclear complex, the pyruvate kinase activity of PKM2 controls the production of pyruvate from PEP, and nuclear PDC further catalyzes the reaction in which pyruvate produces local acetyl-CoA to support the histone acetylation modification at special gene enhancers controlled by p300 (right)

Once mitochondrial activity is suppressed by Bcl-xL (B-cell lymphoma-2-like 1, also known as BCL2L1) overexpression, the levels of citrate and acetyl-CoA are decreased, but there is no obvious decrease in histone H3 or H4 acetylation (Yi et al., 2011). By isolating the nuclear components and confocal microscopy, Sutendra et al. found the presence of PDH, DLAT, and DLD in the nucleus in different types of cells. These components are required for acetyl-CoA generation and the acetylation of the core histones H2B, H3, and H4. Increased nuclear PDC proteins are translocated from mitochondria upon serum stimulation, epidermal growth factor stimulation, or mitochondrial stress during S phase. The inhibition of nuclear PDC by implementing novel strategies decreased the acetylation levels of specific histone lysine residues that are vital for cell cycle progression and S phase entry (de Boer and Houten, 2014; Sutendra et al., 2014) (Fig. 3).

The role of PDC in cancer progression remains inconclusive (Kim et al., 2006; Papandreou et al., 2006; Hitosugi et al., 2011; Kaplon et al., 2013; Sutendra et al., 2014). In mouse and human prostate cancer models, Chen et al. found that mitochondrial PDC provides cytosolic citrate for lipid synthesis, whereas nuclear PDC is critical for the acetylation of H3K9 and the expression of sterol regulatory element-binding transcription factor (SREBF) target genes, such as *ACLY* and squalene epoxidase (*SQLE*). Therefore, PDCs located in different organelles promote lipogenesis and prostate cancer progression by providing substrates and upregulating lipid metabolic enzymes at epigenetically modified levels, respectively (Chen et al., 2018a). The E2 subunit of PDC (also known as DLAT) binds with PKM2 (pyruvate kinase isozyme M2) and p300 to generate a large complex in the nucleus that includes aryl hydrocarbon receptor (AhR), a transcription factor involved in xenobiotic metabolism such as CYP1A1 (cytochrome P4501A1). In this large nuclear complex, the pyruvate kinase activity of PKM2 controls the production of pyruvate from PEP, and nuclear PDC catalyzes pyruvate to produce local acetyl-CoA for histone acetylation at the gene enhancer controlled by p300 (Matsuda et al., 2016) (Fig. 3). A novel oncogene with kinase-domain (NOK), a potent oncogene, promotes histone acetylation by inducing the translocation of PDC from mitochondria to the nucleus, thus causing the occurrence and metastasis of tumors (Shi et al., 2017).

*ACLY* ACLY, which catalyzes the conversion of citrate to acetyl-CoA and OAA, is overexpressed in many cancers and links energy metabolism, biosynthesis, and epigenetic modification (Chypre et al., 2012; Zaidi et al., 2012; Icard et al., 2020). The structural basis for ACLY function was recently revealed (Verschueren et al., 2019). SiRNA knockdown of ACLY or pharmacologic inhibitor SB-204990 inhibiting ACLY activity can significantly increase the mitochondrial membrane potential and inhibit lipid synthesis, cell cycle entry, and cell growth (Hatzivassiliou et al., 2005). By deconvolution microscopy and subcellular fractionation, ACLY was found to exist not only in the cytoplasm but also in the nucleus. Nuclear localized ACLY is the major source of acetyl-CoA accumulation required for histone acetylation and homologous recombination-mediated DNA repair (Wellen et al., 2009; Linder and Mostoslavsky, 2017; Sivanand et al., 2017) (Fig. 3).

During growth factor stimulation or adipocyte differentiation, glucose affects histone acetylation and fatty acid synthesis in an ACLY-dependent manner (Wellen et al., 2009; Lee et al., 2014; Martinez Calejman et al., 2020). The ratio of acetyl-CoA and coenzyme A is glucose-sensitive and determines histone acetylation levels in cancer cells. Activated AKT (AKT serine/threonine kinase) phosphorylates ACLY, resulting in sustained histone acetylation under glucose deprivation conditions, and pAKT (Ser473) was positively correlated with histone acetylation levels in human glioma and prostate cancers (Lee et al., 2014). The AKT-ACLY axis also supports the proliferation of KRAS (Kirsten rat sarcoma 2 viral oncogene homolog)-mutant pancreatic acinar cells, and inhibition of AKT reduces histone acetylation and suppresses acinar-to-ductal metaplasia (ADM). Pancreas-specific deletion of ACLY inhibits ADM and pancreatic tumorigenesis without overt metabolic abnormalities (Carrer et al., 2019). Recently, ACLY was identified as a novel substrate of caspase-10, which is cleaved by caspase-10 at the conserved Asp1026 site. Under metabolic stress conditions, such as glucose starvation, increased caspase-10 downregulates intracellular lipid levels and represses GCN5-mediated histone H3 and H4 acetylation by ACLY cleavage, ultimately inhibiting the expression of tumor-related proliferative genes and metastatic genes as well as tumor progression (Kumari et al., 2019). In patient-derived acute myeloid leukemia (AML) cells, both the substrate and product of phosphoinositide 3-kinase (PI3K), phosphatidylinositol-(4,5)-bisphosphate (PIP_2_), and phosphatidylinositol-(3,4,5)-trisphosphate (PIP_3_), respectively, bind to ACLY. The Src-family kinase (SFK) Lyn directly interacts and phosphorylates the tyrosine residues of ACLY. Inhibitors of PI3K, Lyn, and ACLY action suppress the growth of AML cells by decreasing H3K9 acetylation levels (Basappa et al., 2020).

Macrophage activation or polarization can be finely tuned by metabolic shifts. Upon interleukin-4 (IL-4) stimulation, AKT is activated to enhance glucose utilization in murine bone marrow-derived M2 macrophages. Histone acetylation levels at select M2 genes such as *Arg1*, *Retnla* and *Mgl2*, are increased through AKT-phosphorylated ACLY. SB-204990, the inhibitor of ACLY, indeed suppressed the induction of IL-4/AKT-dependent M2 genes (Covarrubias et al., 2016; Williams and O’Neill, 2018). However, in human monocyte-derived macrophages, ACLY is not required for IL-4-induced macrophage polarization, although pharmacological ACLY inhibitors suppress IL-4-induced target gene expression, suggesting off-target effects of ACLY inhibitors (Namgaladze et al., 2018). It’s known that tumor-associated macrophages (TAMs) create an inflammatory environment that facilitates survival and proliferation of tumor cells, but the role of ACLY-mediated metabolic rewiring of macrophages in tumorigenesis remains unclear. Understanding what conditions within tumors affect the IL-4-AKT-ACLY signaling axis may provide new insights into the role of macrophages in tumor progression. Therefore, tumor microenvironment plays an important role in determining macrophage activity.

Toll-like receptor 4 (TLR4) is an important sensor that recognizes lipopolysaccharide (LPS). Upon LPS recognition, TLR4 promotes the secretion of inflammatory factors and interferon by recruiting four signaling adaptor molecules, including MyD88 (myeloid differentiation primary-response protein 88), MAL (MyD88-adaptor-like protein, also called TIR domain-containing adaptor protein (TIRAP)), TRIF (TIR-domain-containing adaptor protein-inducing IFNB), and TRAM (TRIF-related adaptor molecule). LPS stimulation induces the metabolic reprogramming of glycolysis and the TCA cycle, leading to the accumulation of synthetic citrate and an increase in the acetyl-CoA pool in bone marrow-derived macrophages (BMDMs). MyD88 and TRIF signaling drives LPS-induced ACLY phosphorylation and histone acetylation, and ACLY activation is critical for histone acetylation at the *IL12b* gene locus and for facilitating enhancer chromatin accessibility in response to LPS stimulation (Lauterbach et al., 2019; Williams and O’Neill, 2020). IL-2-induced ACLY phosphorylation and ACLY activation are required for T-cell proliferation, and inhibition of ACLY by SB-204990 induces G_1_/S cell cycle arrest and suppresses the accumulation of histone acetylation levels in IL-2-treated T cells (Osinalde et al., 2016). This study suggests that activation of ACLY in T cells can inhibit tumor growth by promoting the proliferation of T cells.

*ACSS* Glucose-derived pyruvate is the major source of acetyl-CoA generation. In rapidly proliferating cells or hypoxic cells, however, pyruvate preferentially converts to lactate and does not enter mitochondria to produce acetyl-CoA. With findings similar to those showing ACLY-deficient budding yeast reliance on acetate for acetyl-CoA synthesis (De Virgilio et al., 1992; van den Berg et al., 1996; Takahashi et al., 2006), Comerford et al. showed that ACSS2 is the major enzyme required for acetate uptake and utilization and further incorporation into lipids and for histone acetylation in mammalian cells. ACSS2-knockout (KO) reduced the tumorigenesis of hepatocellular carcinoma in a mouse model, and ACSS2 expression was significantly elevated in hepatocellular tumors of mice and in a variety of human tumor samples, including breast, ovarian, and lung cancer tissues, as determined by immunohistochemical (IHC) staining (Comerford et al., 2014). Glucose oxidation contributes less than 50% of the carbon to the acetyl-CoA pool in human brain tumors (Maher et al., 2012), and ^13^C-acetate is oxidized in primary and metastatic mouse glioblastomas (GBMs) *in situ* even with the simultaneous coinfusion of available ^13^C-glucose. ACSS2 expression is required for the incorporation of ^13^C-acetate into glutamate and is positively correlated with the malignancy of GBM (Lyssiotis and Cantley, 2014; Mashimo et al., 2014).

Under metabolic stress, such as hypoxia and lipid-depleted conditions, induced ACSS2 expression promotes the uptake and utilization of acetate to produce acetyl-CoA, which further contributes to fatty acids and supports the biosynthesis of membrane phospholipids. Nuclear-localized ACSS2 maintains the levels of histone acetylation (Schug et al., 2015; Bulusu et al., 2017) (Fig. 3). Exogenous acetate addition rescues the hypoxia-induced decrease in histone acetylation and epigenetically activates lipogenic genes, such as fatty acid synthase (*FASN*) and acetyl-CoA carboxylase 1 (*ACACA*). The high expression of ACSS1 and ACSS2 in hepatocellular carcinoma is critical for acetate-mediated histone acetylation and *de novo* lipogenesis (Gao et al., 2016). AMPK phosphorylates ACSS2 at S659 and promotes its nuclear translocation under glucose deprivation conditions. In the nucleus, ACSS2 binds to transcription factor EB (TFEB) at the promoter regions of lysosomal and autophagy-associated genes and further promotes H3 acetylation and the expression of these genes by locally producing acetyl-CoA from acetate (Li et al., 2017a; Li et al., 2017b). Similar to the yeast system, ACLY-deficient cancer cells primarily use acetate to supply abundant acetyl-CoA by upregulating ACSS2 (Zhao et al., 2016).

Lactate promotes histone acetylation and gene expression in cell culture as an endogenous HDAC inhibitor. Latham et al. found that the effect of lactate, trichostatin A (TSA) and butyrate on gene expression was similar, suggesting that the three of them had a common HDAC inhibition mechanism (Latham et al., 2012). Lactate is known to promote tumorigenesis by providing ATP, acidifying microenvironment, recycling, and immunosuppression. Therefore, the role and contribution of lactate-mediated histone acetylation in tumorigenesis still need further study. Butyrate is a short-chain fatty acid produced by the fermentation of dietary fiber by the gut microbiota in the colon (Scheppach and Weiler, 2004; Hamer et al., 2008). High levels of butyrate in the lumen are the major energy sources that are metabolized to acetyl-CoA by ACLY for the proliferation of normal colonocytes and cancerous colonocytes (Roediger, 1982; Fleming et al., 1991; Donohoe et al., 2012). Butyrate-derived acetyl-CoA induces histone acetylation and regulates gene expression by stimulating HATs and inhibiting HDACs in an ACLY-dependent and ACLY-independent manner, respectively (Donohoe et al., 2012). β-Hydroxybutyrate (β-OHB) is a byproduct of the oxidation of fatty acids. In addition to serving as energetic metabolites, β-OHB has been increasingly shown to promote protein acetylation as a signaling metabolite in two ways. On one hand, the catabolism of β-OHB into acetyl-CoA increases the intracellular acetyl-CoA concentration, which favors the acetylation of histone and nonhistone proteins. On the other hand, under fasting or calorie restriction conditions, endogenous β-OHB binds and inhibits class I histone deacetylase, promotes the acetylation of Lys9 and Lys14 of histone H3 and activates gene transcription controlled by the transcription factor FOXO3a (forkhead box O3A), which is associated with the longevity of a variety of organisms (Shimazu et al., 2013). These findings support the increase in β-OHB concentration observed in mammals during caloric restriction and the resistance of cells to oxidative stress under these conditions. In studies of Drosophila, nematodes, and yeast, class I HDACs have been implicated in the life-extending effects of caloric restriction, suggesting that an environment that increases the β-OHB concentration (e.g., caloric restriction) may extend life by inhibiting class I HDACs.

#### NAD^+^ metabolism and acetylation regulation

NAD^+^ serves as a cofactor of sirtuins during the deacetylation of lysine residues, and it plays important roles in enhancing mitochondrial function and protecting liver and kidney tissues from injury (Katsyuba et al., 2018). NAD^+^ is mainly synthesized from the tryptophan, Preiss-Handler, or nicotinamide (NAM) salvage pathways, with the latter pathway contributing the majority of NAD^+^ (Verdin, 2015; Yang and Sauve, 2016). The NAD^+^/NADH ratio is closely related to the acetylation state and energy state. High glycolytic cells often generate a low NAD^+^/NADH ratio, thereby resulting in the repressed activity of sirtuins, especially SIRT6 which binds NAD^+^ with relatively high affinity (K(d) = 27 ± 1 μmol/L) in the absence of an acetylated substrate (Pan et al., 2011; Madsen et al., 2016). Under stress and nutrient restriction conditions, NAM phosphoribosyltransferase (NAMPT) is induced and protects cells against death induced by genotoxic stress in a SIRT3- and SIRT4-dependent manner (Yang et al., 2007). In Ndufs4 (NADH dehydrogenase [ubiquinone] iron-sulfur protein 4)-KO mice, mitochondrial complex I loss leads to reduced NAD^+^ levels. The addition of nicotinamide mononucleotide (NMN), the precursor of NAD^+^, or cell-permeable α-KG increases the lifespan of Ndufs4-KO mice by promoting protein hyperacetylation (Lee et al., 2019). In addition to affecting acetylation, NAD levels also regulate methylation status. Lozoya et al. found that depletion of mitochondrial DNA (mtDNA) leads to DNA hypermethylation by reprogramming the methionine cycle and increasing SAM levels, almost all of which can be rescued by maintaining mitochondrial NADH oxidation (Lozoya et al., 2018).

#### Acetylation regulates the location, activity and function of transcription factors and metabolic enzymes

Using ^13^C-labeled glucose and gas chromatography mass spectrometry (GC/MS) analysis, the oncogene *c-Myc* was demonstrated to promote fatty acid biosynthesis and H4K16 acetylation by inducing mitochondrial acetyl-CoA generation (Morrish et al., 2010; Edmunds et al., 2014). C-Myc interacts with p300 through its TAD (transcription activation domain), and the Myc-Max complex can be acetylated by p300 and GCN5 (general control of amino acid synthesis 5-like 2 in Yeast). In addition, p300 is recruited by c-Myc to the promoter as a coactivator of the human telomerase reverse transcriptase (*hTERT*) gene to promote transcription (Faiola et al., 2005). Hypoxia-inducible factor 1α (HIF-1α), the master regulator of the hypoxic microenvironment, is acetylated by p300/CBP-associated factor (PCAF) and deacetylated by SIRT1. SIRT1 inhibits HIF-1α activity by blocking p300 recruitment, leading to downregulated glycolysis and retarded tumor growth (Lim et al., 2010).

In addition, the activities of many metabolic enzymes are regulated by acetylation modulation (Choudhary et al., 2009; Zhao et al., 2010). For example, the enzyme activity of glyceraldehyde-3-phosphate dehydrogenase (GAPDH) is increased when it is acetylated at K254 by PCAF acetyltransferase (Li et al., 2014). PCAF-mediated K117 and K251 acetylation of GAPDH is necessary for its nuclear localization after apoptotic stimulation (Ventura et al., 2010). Mitogenic and oncogenic signaling induces p300-mediated acetylation of PKM2 at the K433 residue, promoting PKM2 protein kinase activity and nuclear translocation by preventing its dimer-to-tetramer transition (Lv et al., 2013). Acetylation regulates glucogenesis and PPP by modulating the activity of phosphoenolpyruvate carboxykinase (PEPCK) and 6PGD, respectively (Jiang et al., 2011; Shan et al., 2014). Glucose at high levels stabilize ACLY by inducing PCAF-mediated acetylation at lysine 540, 546, and 554. Acetylated ACLY promotes *de novo* lipid synthesis, cell proliferation, and tumor progression in lung cancer (Lin et al., 2013). Long-chain acyl-CoA dehydrogenase (LCAD), a key mitochondrial fatty acid oxidation enzyme, is a direct target of SIRT3. Hyperacetylation of LCAD at lysine 42 in SIRT3-knockout mice reduced LCAD enzyme activity (Hirschey et al., 2010), and acetylation of lysine residue 318 and 322 of LCAD are two other SIRT3-targeted sites (Bharathi et al., 2013). Branched-chain amino acid transaminase 2 (BCAT2), the rate-limiting enzyme of BCAA metabolism, is acetylated at K44 by CREB-binding protein (CBP). This PTM modulation of BCAT2 promotes its degradation and suppresses BCAA catabolism and pancreatic cancer progression (Lei et al., 2020). Please see Table 1 for more details.

### Metabolic control of (histones) proteins and DNA methylation

#### Writers, readers, and erasers of proteins and DNA methylation

Methylation extensively regulates cellular physiology by modulating the status and activity of proteins (histone) but also of DNA and RNA. Histone methylation ranging from mono- to trimethylation occurs at lysine or arginine residues in H3 and H4 (Di Lorenzo and Bedford, 2011; Kinnaird et al., 2016; Guccione and Richard, 2019). There are only eight residues (H3K4/9/18/23/27/36/79 and H4K20) that undergo significant methylation modulation, but each lysine can support mono-, di-, or trimethylation (Haws et al., 2020). These histone methylation marks can activate or repress gene expression, depending on the types of residues, the number of methyl group(s) added, and the location within the N-terminal regions of H3 or H4 involved (Greer and Shi, 2012). For instance, the methylation of H3K4 and H3K79 is generally associated with transcriptional activation, while H3K9 and H3K27 methylation suppresses gene transcription (Etchegaray and Mostoslavsky, 2016).

Histone methyltransferases (HMTs), as writers of histone methylation, catalyze the methylation reaction in a site-specific manner, mainly on the ε-amino group of lysine residues. Histone demethylases (HDMs) serve as erasers that remove methyl groups from histones. The first identified HDM was lysine-specific histone demethylase 1 (LSD1, also known as KDM1A) in 2004. LSD1 utilizes FAD (also known as vitamin B2) as a cofactor to oxidize the methylated lysine ε-amino to remove methyl groups and produce FADH_2_ (Shi et al., 2004; Anand and Marmorstein, 2007; Greer and Shi, 2012), but LSD1 catalyzes only the removal of methyl groups on mono- or dimethylated lysine residues. Jumonji C (JmjC) domain-containing demethylases (JHDM or Jmj-KDM) are other HDMs that function through a ferrous (Fe^2+^)- and α-KG-dependent dioxygenase mechanisms and are critical for the removal of methyl groups in all three forms (Anand and Marmorstein, 2007; Dimitrova et al., 2015). Methylated lysine residues are recognized by “reader” proteins containing methyl-lysine-binding motifs, including PHD, Tudor, PWWP, WD40, BAH (bromo adjacent homology), ADD (ATRX-DNMT3-DNMT3L), chromodomain (CD), double chromodomain (DCD), tandem Tudor domain (TTD), ankyrinrepeat, MBT (malignant brain tumor), and zn-CW (zinc finger CW) domains. These “reader” proteins have the ability to distinguish target methyl-lysines based on their methylation state and surrounding amino acid sequences (Yun et al., 2011; Musselman et al., 2014; Hyun et al., 2017) (Fig. 2).

In human DNA, the DNA base, especially in CpG islands, can be methylated at the fifth carbon of cytosine (5mC/5-methylcytosine) by DNA methyltransferases (DNMTs), usually resulting in transcriptional repression (Bergman and Cedar, 2013). DNA methyltransferase (writer) enzymes, including DNMT1, DNMT3a, and DNMT3b, are major players in the methylation of 5mC at gene promoters (Greenberg and Bourc’his, 2019). Similar to histone methylation, DNA methylation is also a reversible epigenetic modification. Ten-eleven translocation enzymes TET1, TET2, and TET3 (erasers), which rely on Fe^2+^ and α-KG as co-substrate and cofactor, drive the demethylation of DNA (Pastor et al., 2013). DNA methylation is recognized by methyl-binding proteins (MBPs) (readers), including methyl-CpG-binding protein 2 (MeCP2), MBD1, MBD2, MBD3, MBD4, MBD5, MBD6, SET domain bifurcated 1/2 (SETDB1/2), or bromodomain adjacent to zinc finger domain 2A/B (BAZ2A/B) (Mahmood and Rabbani, 2019) (Fig. 2).

All HMTs and DNMTs require the intermediary metabolite SAM as a methyl donor for both histones and DNA (Takusagawa et al., 1996). Ferrous and α-KG are essential substrates and cofactors for HDMs and TETs. Other TCA cycle intermediary metabolites, such as 2-HG (2-hydroxyglutarate), succinate and fumarate, are also involved in the methylation regulation of histones and DNA by inhibiting HDM and TET activity (Etchegaray and Mostoslavsky, 2016; Haws et al., 2020) (Fig. 2). Although RNA can also be methylated, we focus on discussing how histone and DNA methylation are regulated by SAM derived from one-carbon metabolism (methionine, threonine, and serine metabolism) and other intermediary metabolites derived from the TCA cycle.

#### One-carbon metabolism is directly linked to chromatin dynamics

*S-adenosyl methionine* (*SAM*) *and methylation* Major components of one-carbon metabolism are the methionine cycle and folate cycle. SAM, generated in the methionine cycle, is the primary methyl group donor for histone or DNA methylation (Fig. 2). In mammalian cells, intracellular SAM biosynthesis depends on the condensation of methionine and ATP, which is catalyzed by the rate-limiting enzyme methionine adenosyltransferase Iα or IIα (MATIα or MATIIα) (Sakata et al., 1993; Markham and Pajares, 2009; Reytor et al., 2009). MATIα is expressed specifically in the liver, whereas MATIα is ubiquitously expressed in various tissues (Markham and Pajares, 2009). SAM is demethylated to form S-adenosylhomocysteine (SAH), which is further converted to homocysteine after deadenylation by S-adenosyl homocysteine hydrolase (SAHH). Homocysteine accepts carbon from the folate cycle through 5-methyltetrahydrofolate (mTHF) to generate methionine, resulting in a full turn of the methionine cycle (Locasale, 2013).

SAM is a universal methyl donor and is utilized by methyltransferases to methylate DNA, RNA, metabolites, and proteins, including histones. Methionine metabolism regulates the genomic architecture, chromatin dynamics and gene expression by dynamically modulating trimethylation at lysine 4 on histone H3 (H3K4me3) in both mice with normal physiology and human cancer cells (Dai et al., 2018). Under methionine-limiting conditions, SAM, SAH and the SAM/SAH ratio are dynamically regulated, which reduces the H3K4me3 level and affects the expression of methylation-related enzymes (Mentch et al., 2015). In immortalized mouse embryonic fibroblasts (iMEFs), THP-1 cells, and mouse hepatoma (Hepa-1) cells, MATIIα represses cyclooxygenase 2 (*COX-2*) expression at the mRNA level. Specifically, MATIIα interacts with the histone H3K9 methyltransferase SETDB1, leading to the accumulation of H3K9me3 at the *COX-2* locus and the repression of the *COX-2* gene (Kera et al., 2013). MATIIα also interacts with the transcription factor MafK in the nucleus and acts as a transcriptional corepressor of MafK by affecting the levels of H3K4me2 and H3K9me2. MATIIα is involved in the MafK-mediated suppression of heme oxygenase-1 (HO-1) (Katoh et al., 2011). Moreover, SAM was reported to be involved in innate immunity by regulating H3K4me3 levels in *C*. *elegans* (Ding et al., 2015). Recently, Bian et al. found that tumor cells can absorb a large amount of methionine through the methionine transporter SLC43A2, and competition results in methionine deficiency in T cells, thus affecting epigenetic changes, including loss of H3K79me2 in T cells and impairing the effector function of T cells (Bian et al., 2020).

Glycine N-methyltransferase (GNMT), the most abundant liver methyltransferase, is a SAM-buffering enzyme that catalyzes the transfer of a methyl group from SAM to glycine to form sarcosine, leading to SAM depletion and sarcosine accumulation (Obata et al., 2014; Serefidou et al., 2019). Martinez-Chantar et al. showed that deletion of GNMT in mice induces the hypermethylation of DNA and histones, resulting in steatosis, fibrosis, and hepatocellular carcinoma (Martinez-Chantar et al., 2008). However, Liao et al. found global hypomethylation of DNA in GNMT-knockout mice. In their opinion, decreased DNA methylation is associated with decreased DNMT activity and aberrant DNMT1 and DNMT3b expression (Liao et al., 2009). Hughey et al. also found that elevated SAM promotes polyamine synthesis, polyamine catabolism, transsulfuration, and *de novo* lipogenesis in GNMT-knockout mice (Hughey et al., 2018). Threonine, as the only amino acid critical for the pluripotency of mouse embryonic stem cells (mESCs), regulates stem cell fate by regulating their methylation status. Depletion of threonine from the culture medium or knocking down threonine dehydrogenase (TDH) by shRNAs in mESCs decreased the levels of SAM and H3K4me3, leading to slowed growth and increased differentiation (Shyh-Chang et al., 2013).

Folate is a well-documented metabolite in DNA methylation (Crider et al., 2012; Ly et al., 2012). Diets low in folate cause genomic DNA hypomethylation, which can affect DNA stability and gene expression and increase the risk of neoplasia. Physiological intake of folic acid can reverse this phenomenon in patients with colorectal adenocarcinoma (Pufulete et al., 2005). Folate supplementation effectively decreased the degree of DNA hypomethylation of the rectal mucosa, but only in patients with a single polyp (Cravo et al., 1998). Similar to many other metabolites, folate can be detected in the nucleus (Zamierowski and Wagner, 1977). In the nucleus, folate is bound to LSD1 and protects LSD1 from inhibition by formaldehyde (Luka et al., 2011; Luka et al., 2014). In mice treated with a folate-deficient diet, reduced folate levels in the liver are associated with increased methylated H3K4 levels due to decreased LSD1 activity (Garcia et al., 2016).

*Serine and glycine metabolism in methylation regulation* Serine and glycine, which are involved in nucleotide synthesis, methylation reactions, and the generation of GSH (glutathione) and NADPH (the reduced form of nicotinamide adenine dinucleotide phosphate), are additional important one-carbon donors that are integrated with the folate cycle. In most cultured cells, serine donates its β-carbon atom to tetrahydrofolate (THF) via serine hydroxymethyltransferases (SHMTs), generating glycine and 5,10-methylene-THF (me-THF), which initiates the folate cycle. Serine and glycine regulate methylation by linking with the folate cycle, which is coupled to the methionine cycle (Fig. 2). me-THF can also be produced by the glycine cleavage system (glycine dehydrogenase (GLDC) is the major component), in which glycine is cleaved into ammonia, carbon dioxide, and a carbon unit, which is involved in the methylation of THF (Locasale, 2013; Yang and Vousden, 2016). Moreover, other nutrient sources, including threonine, choline, betaine, dimethylglycine, and sarcosine (N-methylglycine), regulate methylation reactions via their convertion into glycine (Wang et al., 2009; Locasale, 2013).

Serine can provide one-carbon units to generate methionine from homocysteine; in addition, ATP (purine) generated by serine-mediated *de novo* synthesis is also involved in the production of SAM from methionine (Fig. 2). In colorectal cancer cells, methionine is the major methyl donor, and serine does not directly provide one-carbon units for methylation under conditions of methionine supplementation. However, serine availability controls the methyl transfer from methionine to DNA and RNA because this process is impeded during serine starvation. In brief, serine-contributed ATP synthesized *de novo* (based on serine availability) is critical for the SAM cycle regardless of whether methionine is present, and the role of serine is highlighted in supporting DNA/RNA methylation through the maintenance of nucleotide levels (Maddocks et al., 2016; Parker and Metallo, 2016). The serine-responsive SAM-containing metabolic enzyme complex (SESAME) is a supercomplex consisting of pyruvate kinase, serine metabolic enzyme, and SAM synthetases in yeast. The interaction of SESAME with the Set1 H3K4 methyltransferase complex regulates H3K4 methylation and H3T11 phosphorylation (H3pT11) by sensing glycolysis and glucose-derived serine metabolism (Li et al., 2015b). LKB1 (also known as STK11) loss and KRAS activation (KRAS^G12D^) synergistically potentiate glycolysis, serine metabolism, and tumorigenesis. In LKB1-deficient cells, the activated *de novo* serine biosynthesis pathway promotes DNA methylation. LKB1 loss decreases phosphoserine aminotransferase 1 (PSAT1)-mediated DNA methylation and retrotransposon expression, important modulators of host gene expression. Tumor-bearing mice with LKB1 loss and human LKB1-mutant pancreatic tumor cells are more sensitized to DNMT knockdown or DNMT inhibitor decitabine treatment, which inhibits serine biosynthesis and DNA methylation (Kottakis et al., 2016). More recently, another serine biosynthesis enzyme, SHMT2, was reported to initiate lymphoma development by epigenetically silencing tumor suppressors. The *SHMT2* gene is amplified in human B cells. Elevated *SHMT2* expression in human and mouse follicular lymphoma (FL), the most common form of B-cell lymphoma, is controlled by MYC, and a similar mechanism has been reported in hepatoma carcinoma cells (Sun et al., 2015). SHMT2 activation induces SAM synthesis to promote DNA and histone methylation, leading to promoter silencing of previously unappreciated tumor suppressor genes, such as SAM and SH3 domain-containing protein 1 (*SASH1*) and protein tyrosine phosphatase receptor type M (*PTPRM*), and the initiation of lymphomagenesis (Parsa et al., 2020).

#### TCA cycle-derived intermediary metabolites regulate methylation status

*α-KG regulates histone and DNA methylation* Although the TCA cycle is known to play central roles in ATP production, it is also now appreciated as a source of biosynthetic precursors and chemical intermediates (DeBerardinis et al., 2008a). α-KG, also known as 2-oxoglutarate (2-OG), is generated from isocitrate in a reaction catalyzed by cytoplasmic IDH1 (isocitrate dehydrogenase 1) or mitochondrial IDH2 and IDH3, accompanied by the production of NADPH from NADP. α-KG is a cosubstrate required for the histone demethylase JHDM and DNA demethylase TETs, as described above (Fig. 2). In addition to isocitrate, other amino acids, such as arginine, histidine, proline, and glutamate from glutamine-derived glutaminolysis also mediate α-KG synthesis (Wise et al., 2011; Metallo et al., 2012; Mullen et al., 2012; Kaelin and McKnight, 2013). The core region of solid tumors, such as melanoma and breast cancer, displayed low glutamine levels compared with the tumor periphery, as determined by liquid chromatography-mass spectrometry (LC-MS) analysis. In patient-derived ^V600E^*BRAF* melanoma cells, treatment to ensure low glutamine levels significantly decreased α-KG levels, which led to the hypermethylation of histone H3, H3K27-mediated tumor dedifferentiation, and resistance to BRAF inhibitor treatment. Knocking down the H3K27-specific demethylase KDM6B mimics the low-glutamine condition and mediates resistance to PLX4032 (BRAF inhibitor) treatment, and the opposite results are obtained when H3K27 methyltransferase EZH2 is knocked down (Pan et al., 2016). Epigenetic and metabolic reprogramming coordinates the polarization of macrophages and contributes to their functional plasticity (Ivashkiv, 2013; O’Neill and Pearce, 2016). Glutamine-derived α-KG is also important for the alternative (M2) activation of macrophages. A high α-KG/succinate ratio is found in IL-4-induced M2 macrophages compared to LPS-induced M1 macrophages. M2 polarization depends on the α-KG–JMJD3-mediated demethylation of H3K27 (Liu et al., 2017). Moreover, intracellular α-KG derived from glucose or glutamine promotes H3K27 demethylation and TET-dependent DNA demethylation, contributing to the maintenance of embryonic stem cell (ESC) pluripotency (Carey et al., 2015). PSAT1, a serine biosynthesis transaminase, mediates the production of α-KG. PSAT1 knockdown is sufficient to reduce intracellular α-KG and accelerate the differentiation of mouse ESCs by modulating DNA 5’-hydroxymethylcytosine (5’-hmC) and histone methylation levels (Hwang et al., 2016).

*IDH mutation-induced 2HG regulates DNA and histone methylation* Two independent groups undertaking cancer genome sequencing projects identified *IDH1* mutations in both glioblastoma multiforme and acute myeloid leukemia in 2008 and 2009, respectively (Parsons et al., 2008; Mardis et al., 2009). A missense mutation in a single arginine residue, R132, in the enzyme active site is sufficient to cause IDH1-related disease alteration. Mutations in IDH2 are also apparent in GBM and other cancers (Yan et al., 2009). The R132H substitution of IDH1 (Parsons et al., 2008; Mardis et al., 2009) and the R172K and R140Q substitutions of IDH2 (Ward et al., 2010) constitute the majority of mutational events and lead to the occurrence of GBM, AML, chondrosarcoma, cholangiocarcinoma, and angioimmunoblastic T-cell lymphoma (Cairns and Mak, 2013; Lu et al., 2013). Mutant IDH1 and IDH2 are oncogenes that catalyze the conversion of α-KG to 2HG in an NADPH-dependent manner (Dang et al., 2009; Losman and Kaelin, 2013). There are two enantiomeric forms of 2HG, D-(or R-) and L-(or S-) type 2-HG, all of which are α-KG inhibitors that inhibit α-KG-dependent histone lysine demethylases, such as FIH (factor inhibiting HIF), PHD2 (prolyl hydroxylase domain-containing protein, also known as HIF prolyl-hydroxylase 2), and JMJDs (Chowdhury et al., 2011).

D2HG is the major form in diseases with IDH1 or IDH2 mutants (Dang et al., 2009; Gross et al., 2010). FAD-dependent D-2-hydroxyglutarate dehydrogenase (D2HGDH) regulates the generation of D2HG in *E*. *coli*, yeast, and human cancer cells (Zhao and Winkler, 1996; Fan et al., 2015; Lin et al., 2015; Becker-Kettern et al., 2016; Ye et al., 2018). Leukemic IDH1 and IDH2 mutants induce global DNA hypermethylation, destroy TET2 function, impair hematopoietic differentiation, increase the expression of stem/progenitor cell markers, and ultimately promote malignant transformation (Figueroa et al., 2010). In nontransformed cells, adipocytes, and immortalized astrocytes, the introduction of either mutant IDH or cell-permeable 2HG blocks cell differentiation by inducing global and promoter-specific H3K9 and H3K27 methylation (Lu et al., 2012).

*D2HG regulates the HIF-1 signaling axis* HIF-1 protein levels are precisely controlled by PHDs, also known as Eglnine homologs (EGLNs), which are α-KG-dependent dioxygenases that function as cellular oxygen sensors. The R132H mutant of tumor-derived IDH1 showed decreased catalytic activity due to impaired isocitrate binding and reduced α-KG levels, leading to elevated HIF-1α protein levels in human glioblastoma cells (Zhao et al., 2009). An increase in HIF in IDH-mutant tumors is usually present in necrotic areas and is presumed to be due to severe hypoxia (Williams et al., 2011). Losman et al. found that D2HG, but not L2HG, promotes leukemic transformation in a dose- and passage-dependent manner. In TF-1 human erythroleukemia cells overexpressing the IDH1 R132H mutant, HIF-1α is diminished due to the agonistic effect of D2HG on PHDs (Losman et al., 2013; McCarthy, 2013; Ye et al., 2013). In immortalized human astrocytes and HCT116 colorectal cancer cells, D2HG stimulates PHD activity by acting as its cosubstrate, resulting in reduced HIF levels and ultimately enhancing cell proliferation and transformation (Koivunen et al., 2012). This regulatory complexity indicates that D2HG-regulated HIF stability is cell type- and context-dependent (Losman and Kaelin, 2013).

*The roles of L2HG in tumor cells and immune cells* In renal cell carcinoma (RCC), accumulated L2HG mediates epigenetic modifications by serving as an oncometabolite and an epigenetic modifier. Lower expression of L-2-hydroxyglutarate dehydrogenase (L2HGDH) in RCC results in the accumulation of L2HG and reduces 5hmC levels on DNA. This outcome is consistent with the 2HG-mediated suppression of TET enzymes, which convert 5mC to 5hmC. The re-expression of L2HGDH promotes 5hmC accumulation, reduces H3K27me3 and H3K9me3 levels, and inhibits the proliferation of RCC cells (Shim et al., 2014). Moreover, enhanced L2HG production is also found under hypoxic conditions (Intlekofer et al., 2015; Oldham et al., 2015), and in turn, L2HG stabilizes HIF-1 protein levels by inhibiting PHD activity (Koivunen et al., 2012). ^13^C-labeled glucose or glutamine assays demonstrated that glutamine-derived α-KG is critical for hypoxia-induced L2HG generation. Although IDH controls the generation of D2HG, knocking down IDH1 or IDH2 did not affect L2HG levels in response to hypoxia. L2HG levels are modestly decreased by knocking down MDH1 or MDH2 (malate dehydrogenase), which are known to convert α-KG to L2HG (Rzem et al., 2007), but knocking down LDHA (lactate dehydrogenase A) strikingly decreased L2HG accumulation in hypoxic cells (Intlekofer et al., 2015). L2HG accumulation is necessary and sufficient for the activation of H3K9me3 and repressive histone methylation (Intlekofer et al., 2015) and inhibits electron transport and glycolysis to alleviate reductive stress (Oldham et al., 2015). In response to T-cell receptor triggering, the accumulation of L2HG in mouse CD8^+^ T cells depends on the VHL-HIF-LDHA axis and PDK-PDH signaling. In turn, L2HG stabilizes HIF-1α and modulates the global histone H3K27me3. L2HG induction or supplementation enhances the proliferation, long-term persistence and antitumor capacity of adoptively transferred CD8^+^ T cells (Tyrakis et al., 2016; Cairns and Mak, 2017).

*Fumarate and succinate antagonize the roles of α-KG* In addition to *IDH1* and *IDH2*, germinal and somatic mutations of fumarate hydratase (*FH*) and succinate dehydrogenases (*SDHA*, *SDHB*, *SDHC*, *SDHD*, and *SDHAF2*), encoding FH and SDH enzymes, are common in a number of human cancers (Baysal et al., 2000; Astuti et al., 2001; Hao et al., 2009; Kaelin, 2009; Bayley et al., 2010; Oermann et al., 2012). Accumulated fumarate and succinate resulting from *FH* and *SDH* mutations share structural similarity with α-KG. Both fumarate and succinate increase global histone methylation and HIF-1α protein levels and reduce endostatin in cultured cells by inhibiting the activity of α-KG-dependent KDMs. In addition, TET-mediated 5hmC production is decreased by knocking down FH or SDH or supplementation with fumarate or succinate. These epigenetic alterations induced by *FH* or *SDH* loss contribute to tumorigenesis (Xiao et al., 2012). The epithelial-to-mesenchymal transition (EMT) has been implicated in tumor progression and metastasis. In human FH-deficient UOK262 cells, mesenchymal markers, including *Snai2*, *Zeb1* and *Zeb2,* are induced, and the re-expression of FH reverses the expression of these markers. Fumarate inhibits the TET-mediated demethylation of anti-metastatic miR-200, leading to the induction of EMT-related transcription factors and enhanced migratory potential of renal cancer (Sciacovelli et al., 2016).

*Oncometabolites hinder DNA repair* Recently, two studies by Sulkowsk et al. suggested that IDH, FH, or SDH mutation-induced accumulation of 2-HG, fumarate, or succinate suppresses the homologous recombination (HR) DNA repair pathway in gliomas and AML with mutant IDH, hereditary leiomyomatosis and renal cell cancer (HLRCC), and succinate dehydrogenase-related hereditary paraganglioma and pheochromocytoma (SDH PGL/PCC) (Sulkowski et al., 2017; Sulkowski et al., 2018). In 2020, Sulkowsk et al. further revealed the pathways in which metabolites (2HG, succinate, and fumarate) interfere with DNA repair. By inhibiting the activity of the histone demethylase KDM4B, tumor cell metabolites cause the hypermethylation of H3K9me3 at DNA break sites, thus affecting DNA homology-dependent repair (HDR). Subsequently, the enrichment of key HDR molecules TIP60 (tat-interacting protein, also known as histone acetyltransferase KAT5) and ATM (ataxia telangiectasia mutated) and downstream repair factors at DNA fracture sites was reduced. This oncometabolite-induced HDR defect confers intensive sensitivity to poly (ADP-ribose) polymerase (PARP) inhibitors being tested in clinical trials (Chen and Xiong, 2020; Sulkowski et al., 2020). Therefore, this study explains a molecular mechanism of tumor metabolite-induced HDR inhibition and suggests a potential therapeutic strategy for tumor therapy.

### Succinyl-CoA and (histone) succinylation

Research on succinylation stemmed from its role in inhibiting antibody formation and testing allergic skin responses in animals that were sensitive to dinitrophenyl-polyline in 1962 (Parker et al., 1962). In the following years, the succinylation of pepsinogen (Gounaris and Perlmann, 1967), ovalbumin (Kidwai et al., 1976), and histone amino groups (Pineiro et al., 1992) was studied in succession. However, it was not until 2011 that succinylation was identified as a natural PTM of lysine residues in bacteria by affinity purification with anti-succinyl lysine antibody (Zhang et al., 2011; Alleyn et al., 2018; Sreedhar et al., 2020). Succinylation of lysine (Ksucc) residues converts the cationic lysine side chain into an anionic chain with large potential impacts on protein structures, charges, and functions, and this modification is reversible, dynamic, and evolutionarily conserved in both prokaryotes and eukaryotes (Xie et al., 2012; Weinert et al., 2013; Wang et al., 2017b; Kurmi et al., 2018; Wang et al., 2019a).

TCA cycle-derived succinyl-CoA is the major substrate for succinylation. Succinyl-CoA can be generated from the TCA cycle, lipids, and amino acid metabolism (histidine, proline, glutamine, glutamate, methionine, and the BCAAs isoleucine, leucine, and valine) (Hirschey and Zhao, 2015) and then synthesized by succinyl-CoA synthetase. As early as 1992, Pineiro et al. noticed that the transcriptional properties of succinylated nucleosomal cores are similar to those of acetylated particles, which had been observed in 1991 (Pineiro et al., 1991; Pineiro et al., 1992). Defects in the TCA cycle by the depletion of SDH increase succinyl-CoA, and subsequent histone hypersuccinylation correlates with active gene expression (Smestad et al., 2018). The α-ketoglutarate dehydrogenase complex (α-KGDH) in the nucleus can bind to lysine acetyl transferase 2A (KAT2A) in gene promoter regions, and KAT2A binds to succinyl CoA and acts as a succinyltransferase to succinylate histone H3 on lysine 79. If the α-KGDH complex is blocked from entering the nucleus or KAT2A protein expression is inhibited, the expression of downstream target genes can be reduced, thus inhibiting tumor growth (Wang et al., 2017b; Wang et al., 2018b; Xu et al., 2021). Moreover, carnitine palmitoyltransferase 1A (CPT1A) is found to have lysine succinyltransferase activity upon the succinylation of S100A10 in gastric cancer (Kurmi et al., 2018; Wang et al., 2019a). These studies showed that the nonmetabolic functions of α-KGDH and CPT1A play important roles in tumor progression. Although succinyl-CoA is mainly synthesized in mitochondria, cytosolic succinate is converted back to succinyl-CoA (Alarcon et al., 2002), a result that reasonably explains how proteins undergo lysine succinylation in the cytoplasm and nucleus.

KAT2A and CPT1A are writers of protein succinylation, and SIRT5 has been identified as an eraser of Ksucc by catalyzing the hydrolysis of succinyl lysine *in vitro* and desuccinylating several mammalian proteins, such as glutamate dehydrogenase (GDH), malate dehydrogenase, and citrate synthase (CS), that were identified by mass spectrometry to have been modified by succinylation (Du et al., 2011) (Fig. 2). SIRT7 was identified as another histone desuccinylase, especially in response to DNA damage (Li et al., 2016). The YEATS domain of GAS41 recognizes succinylation as a pH-dependent reader of Ksucc (Wang et al., 2018a). Park et al. identified 2,565 succinylation sites on 779 proteins regulated by SIRT5 in mammalian fibroblasts and liver tissues, and found that diverse mitochondrial and nonmitochondrial metabolic enzymes can be succinylated (Park et al., 2013). Most of the 2,565 succinylation sites do not overlap with acetylation sites; however, thousands of succinylation sites mapped by Weinert et al. in diverse organisms including bacteria, yeast, and human cells, extensively overlap with acetylation sites (Weinert et al., 2013). SIRT5 is a mitochondrial protein, and in SIRT5-KO mice, the mitochondrial lysine succinylome in liver tissues is significantly changed. The metabolic pathways of fatty acid β-oxidation and ketogenesis are highly targeted by SIRT5. Lack of SIRT5 impairs β-oxidation and promotes the accumulation of acylcarnitines. 3-Hydroxy-3-methylglutaryl-CoA synthase 2 (HMGCS2), the rate-limiting enzyme of ketogenesis, is hypersuccinylated in the absence of SIRT5, but this modification of HMGCS2 inhibits its activity and reduces ketone body production (Rardin et al., 2013). IDH1 mutant-induced production of 2-HG inhibits SDH activity and promotes hypersuccinylation in mitochondria, which induces cancerous metabolism and apoptosis resistance (Li et al., 2015a). These results demonstrate that SIRT5 is a global regulator of lysine succinylation in the cytosol, mitochondria, and nucleus and indicate mechanisms for preventing tumorigenesis by modulating protein succinylation (Park et al., 2013; Rardin et al., 2013; Stram and Payne, 2016; Carrico et al., 2018).

PKM2, a notable metabolic enzyme, is succinylated at lysine 498 (K498), which increases PKM2 activity and sensitizes cells to oxidative damage by decreasing cellular NADPH production. These processes are reversed by SIRT5, which is critical for PKM2 desuccinylation by binding to PKM2 at K498 (Xiangyun et al., 2017). However, under nutrient stress conditions, such as glucose starvation, K433 succinylation of PKM2 promotes its translocation to mitochondria, whereas succinylation of K498 has no effect on this process. Mitochondrial PKM2 prevents the degradation of voltage-dependent anion channel 3 (VDAC3) and increases mitochondrial permeability to generate more ATP for cell survival under glucose starvation conditions (Qi et al., 2019). In addition, UCP1 (mitochondrial uncoupling protein 1) is a novel target of SIRT5-mediated desuccinylation recently identified in brown fat tissues in SIRT5-KO mice (Wang et al., 2019b). ACOX1 (acyl-CoA oxidase 1) and IDH2 are desuccinylated by SIRT5 in response to oxidative damage (Zhou et al., 2016; Chen et al., 2018b). SIRT5 also inhibits ubiquitin-mediated glutaminase (GLS) degradation by desuccinylating glutaminase, thereby regulating mitophagy and tumorigenesis (Polletta et al., 2015; Greene et al., 2019).

SDHA, which mediates succinate dehydrogenation, is a direct target of SIRT5, and desuccinylation of SDHA by SIRT5 suppresses SDH activity and cellular respiration (Park et al., 2013). Succinate has been shown to act as a proinflammatory metabolite that accumulates in LPS- or interferon-γ (IFN-γ)-treated macrophages (Tannahill et al., 2013; Jha et al., 2015). LPS-induced glutamine-derived succinate stabilizes HIF-1α protein expression, which is critical for LPS-induced interleukin-1β (IL-1β) transcription. A twofold increase in protein succinylation, such as MDH, was discovered in LPS-treated macrophages, a result that can be explained by an increase in succinate and a decrease in the expression of desuccinylase SIRT5 in macrophages (Tannahill et al., 2013). Analogous to its status in tumors, PKM2 is succinylated in macrophages. The succinylation of PKM2 at K311 inhibits PKM2 enzyme activity and promotes its nuclear translocation in dimer form. In the nucleus, by performing nonmetabolic functions, the dimeric form of PKM2 binds to HIF-1α to promote the transcription of IL-1β. Desuccinylation of PKM2 by SIRT5 blocks LPS-induced IL-1β expression to prevent DSS (dextran sodium sulfate)-induced colitis in mice (Wang et al., 2017a).

### Ketone body-derived β-hydroxybutyrate modulates protein hydroxybutyrylation

Ketone bodies contain three different molecules, acetone, acetoacetate (AcAc), and β-OHB, which are byproducts of the oxidation of fatty acids in the liver to provide energy for the heart and brain under fasting conditions (Newman and Verdin, 2014a, b, 2017). In addition to serving as energy metabolites and promoting protein acetylation, as mentioned above, Xie et al. discovered a novel epigenetic modification, histone lysine β-hydroxybutyrylation (Kbhb), which is closely related to ketone body metabolism (Fig. 2). The researchers found significant increases in histone lysine β-hydroxybutyrylation modification in mouse liver cells but no change in acetylation modification that is mainly derived from glucose metabolism under fasting conditions. Further ChIP-qPCR (chromatin immunoprecipitation (ChIP) coupled with quantitative PCR) assays and ChIP-seq data showed that with the increase in histone Kbhb modification, the expression of some genes related to physiological responses to fasting was upregulated, such as amino acid catabolism, redox balance, circadian rhythm, and PPAR (peroxisome proliferator-activated receptor) signaling (Xie et al., 2016). Histone acyltransferase p300 acts as a writer to mediate histone β-hydroxybutyrylation (Kaczmarska et al., 2017), and human SIRT3 acts as an eraser to selectively remove histone β-hydroxybutyrylation with a preference for H3K4, K9, K18, K23, K27, and H4K16 but has no activity with H4K5, K8, and K12, which distinguishes it from Zn-dependent class I HDACs (Abmayr and Workman, 2019; Zhang et al., 2019b). The Kbhb levels of Lys9 in histone H3 (H3K9bhb) were reduced in the brains of depressed mice, and exogenous β-OHB rescued this phenomenon and ameliorated depressive behaviors of these mice (Chen et al., 2017).

Naïve CD8^+^ T (T_n_) cells can differentiate into CD8^+^ effector T (T_eff_) cells after receiving antigen stimulation. Some T_eff_ cells persist and develop into long-lived CD8^+^ memory T (T_mem_) cells. Fatty acid-mediated oxidative phosphorylation and phosphoenolpyruvate carboxykinase 1 (PCK1)-mediated glycogen metabolism control the formation and maintenance of CD8^+^ T_mem_ cells (Pearce et al., 2009; Ma et al., 2018). Recently, Zhang et al. found that acetyl-CoA in mitochondria indirectly regulates PCK1 expression through the ketogenesis pathway, and epigenetic modification of histones through β-OHB is also critical for the formation of CD8^+^ T_mem_ cells (Zhang et al., 2020). Accumulated β-OHB and AcAc, but not acetone, were found in CD8^+^ T_mem_ cells compared to T_n_ or T_eff_ cells, as determined by LC-MS analysis. After β-OHB and AcAc treatment of induced T cells, it was found that only β-OHB was specifically involved in the memory formation of CD8^+^ T_mem_ cells, and similar results were observed in mice treated with a carbohydrate-free ketogenic diet. Furthermore, β-OHB facilitates CD8^+^ T_mem_ cell formation by upregulating H3K9bhb of *Foxo1* and *Ppargc1a* (which encodes PGC-1α), two transcription factors critical for *Pck1* expression. These results provide new insights into how CD8^+^ T_mem_ cells balance energy supply and redox homeostasis and into their potential clinical applications for regulating T cell memory.

In addition to the hydroxybutyrylation modification of histones, p53 can undergo hydroxybutyrylation modification in the presence of β-OHB, and this modification is catalyzed by histone acetyltransferase CBP/p300 (Kaczmarska et al., 2017).This modification of p53 was apparent in cells treated with β-OHB and in thymic tissues of mice whose fasting resulted in increased serum β-OHB concentration. Hydroxybutyrylation of p53 inhibits its acetylation and reduces the expression of its target genes, including *p21* and *PUMA*, ultimately promoting cell proliferation (Liu et al., 2019).

### Lactylation is a novel PTM mediated by lactate and plays roles in addition to lactate metabolism

Lactic acid has long been considered a waste product of glycolytic metabolism; however, in 2017, two independent groups recently found that it can be reused as the primary carbon source for the mitochondrial TCA cycle in normal tissues and tumors (Faubert et al., 2017; Hui et al., 2017; Sun et al., 2018). Recently, it was found that lactic acid is integrated into cell metabolism by inducing dynamic endoplasmic reticulum-mitochondrial Mg^2+^ changes (Daw et al., 2020). Lactate was also found to promote histone acetylation and regulate gene expression as an HDAC inhibitor (Latham et al., 2012). Furthermore, inspired by the wide acylation of histones by intracellular metabolites, Zhang et al. recently found that lactic acid can also modify the lysine residues of histones in a new epigenetic modification known as lactylation (Zhang et al., 2019a) (Fig. 2). Twenty-eight lysine lactylation (Kla) sites on core histones, including H3, H4, H2A, and H2B, were identified in human HeLa cells and mouse BMDMs. The lactylation of H3 and H4 is p53-dependent and p300-mediated (Fig. 2). Hypoxia and macrophage polarization, which are associated with increased lactate derived from activated glycolysis, can enhance intracellular histone lactylation. In the late phase of M1 macrophage polarization, increased histone Kla directly promotes gene transcription and induces homeostatic genes, including *Arg1*, a marker of M2 macrophages. Interestingly, the researchers also detected histone lactylation in macrophages isolated from mouse melanoma and lung tumors and observed a positive correlation between histone lactylation and oncogenic production by reparative M2 macrophages. These findings suggest that high lactate and histone lactylation levels in macrophages may contribute to tumor formation and progression.

It is surprising that a single metabolite can have such a powerful effect on immune cell function. The discovery of histone lactylation and its impact on macrophage biology is a blueprint for understanding how lactic acid changes other cell types, unlocking the mysteries of the Warburg effect and understanding its impact on human disease. Whether cancer cells and other immune cells, such as T cells, can be regulated through this mechanism is unclear. In addition to cancer, the Warburg effect has been observed in other diseases, including sepsis, autoimmune diseases, atherosclerosis, diabetes, and aging. Therefore, more studies are needed on the role and regulation of this newly discovered histone modification.

### ATP-, *O*-GlcNAc-, citrulline-, and itaconate-mediated phosphorylation, *O*-GlcNAcylation, citrullination, and itaconation

#### ATP and phosphorylation

In addition to lysine and arginine, serine and threonine can be extensively modified, such as by phosphorylation and *O*-GlcNAcylation. Histone phosphorylation is a dynamic modification in which phosphate groups are added to residues of serine or threonine. Phosphorylation was first discovered in the late 1960s (Kleinsmith et al., 1966; Gutierrez and Hnilica, 1967), and the first histone kinase was discovered in 1968 (Langan, 1968). Only these serine and threonine residues have established as residues for histone phosphorylation, but subsequent data suggest that histone tyrosine residues can also be phosphorylated (Cook et al., 2009; Dawson et al., 2009; Singh et al., 2009b; Xiao et al., 2009). AMPK is a sensor of cellular energy status consistent with the ratios of AMP:ATP and ADP:ATP, and this kinase is activated by an increase in AMP or ADP but inactivated by ATP (Hardie, 2011; Hardie et al., 2016). Yeast AMPK homolog Snf1 kinase is required for the phosphorylation of histone H3 at serine 10 (H3pS10) in the promoter of the *INO1* gene (Lo et al., 2001). Mammalian AMPK was reported to phosphorylate histone H2B at serine 36 (H2BpS36) (Bungard et al., 2010) (Fig. 2). As a nutrient sensor, AMPK can be activated under a variety of stress conditions. During glucose deprivation, AMPK is localized to the promoter of p53 and activates p53-responsive genes, which are essential for cell survival under metabolic stress. Currently, an increasing number of studies have recognized the importance of AMPK in tumorigenesis; therefore, methods to activate AMPK activity, such as exercise, calorie restriction and metformin treatment, have been identified, and some have been tested in preclinical models (Steinberg and Carling, 2019).

Histone phosphorylation is associated with many different cellular processes, such as transcriptional activation, mitosis, meiosis, DNA repair, and apoptosis (Cohen et al., 2011). Although all histones H1, H2A, H2B, H3, and H4 can be phosphorylated at multiple sites, H3 phosphorylation has been most widely and intensively studied (Prigent and Dimitrov, 2003; Nowak and Corces, 2004). H3S10 can be phosphorylated by a variety of kinases, such as mitogen- and stress-activated protein kinase 1 and 2 (MSK1 and MSK2) (Soloaga et al., 2003), ribosomal S6 kinase 2 (RSK2) (Sassone-Corsi et al., 1999), I κB kinase-α (IKK-α) (Anest et al., 2003), and PIM1 kinase (Zippo et al., 2007). PIM1 kinase-mediated H3S10 phosphorylation at the E-box of Myc target genes is involved in gene activation and contributes to cell transformation and tumor growth (Zippo et al., 2007). The overexpression of histone H3 enhanced the neoplastic cell transformation induced by epidermal growth factor and cancer development, and histone H3 phosphorylation-mediated c-*jun* and c-*fos* induction is critical for these processes (Choi et al., 2005). The phosphorylation of H3S10 is a hallmark of mitosis, starting in prophase, reaching its highest level in metaphase, and then decreasing toward the end of the cell cycle. Mitotic H3S10 phosphorylation is mainly controlled by Aurora B (Adams et al., 2001; Giet and Glover, 2001; Richie and Golden, 2005), Aurora A (Kim et al., 2016), and polo-like kinase 1 (PLK1). Moreover, histone phosphorylation regulates tumorigenesis by participating in DNA damage repair and apoptosis.

#### O-GlcNAc and O-GlcNAcylation

*O*-linked β-N-acetylglucosamine glycosylation (*O*-GlcNAcylation) is a PTM that regulates basic cellular processes. UDP-GlcNAc (uridine diphosphate GlcNAc), the final product of the hexosamine biosynthetic pathway (HBP) that integrates glucose, glutamine, amino acid, fatty acid, and nucleotide metabolism, serves as the donor substrate for *O*-GlcNAcylation (Hart et al., 2007; Hart et al., 2011; Ferrer et al., 2016; Yang and Qian, 2017) (Fig. 2). In contrast to other PTMs, which are regulated by many writers and erasers, glycosylation is regulated by a single pair of enzymes, *O*-GlcNAc transferase (OGT) and *O*-GlcNAcase (OGA). OGT catalyzes the transfer of GlcNAc from UDP-GlcNAc to the Ser and Thr residues of target proteins, whereas OGA catalyzes the hydrolysis of this PTM (Hart et al., 2007; Hart et al., 2011; Slawson and Hart, 2011) (Fig. 2). In addition to relying on nutrient metabolism to provide a substrate, *O*-GlcNAcylation is sensitive to various types of cellular stresses, such as heat shock, hypoxia, and nutrient deprivation (Hart et al., 2011; Ferrer et al., 2014; Yang and Qian, 2017). However, the detailed mechanism by which cells sense these stresses to induce *O*-GlcNAcylation and how various stimuli trigger dynamic changes in protein *O*-GlcNAcylation are far from clear. After heat shock, the *O*-GlcNAcylation of histones increases rapidly, and this increase is correlated with an increase in DNA compaction (Sakabe et al., 2010). By modifying proteins such as nuclear factor-κB (NF-κB), nuclear factor of activated T cells cytoplasmic 1 (NFATC1) (Golks et al., 2007), CREB-regulated transcription coactivator 2 (CRTC2) (Dentin et al., 2008), forkhead box O1 (FOXO1) (Housley et al., 2008), PPARG coactivator 1α (PGC1α) (Ruan et al., 2012), and RNA polymerase II (Pol II) (Kelly et al., 1993) or by proteins interacting with HDAC (Yang et al., 2002), EZH2 (Chu et al., 2014), PRC2 (Gambetta et al., 2009), or TET2 (Dehennaut et al., 2014; Lewis and Hanover, 2014; Singh et al., 2015), *O*-GlcNAcylation regulates fundamental cellular processes such as transcription, epigenetic programming, and cell signaling pathway activation (Hardiville and Hart, 2014; Hart, 2019). Therefore, in view of its importance in cellular processes, the disruption of *O*-GlcNAcylation is related to the pathological processes of many kinds of tumors. Several studies showed decreased *O*-GlcNAc levels in some tumor samples compared with matched adjacent tissues (Slawson et al., 2001), whereas other studies found accumulated *O*-GlcNAc, enhanced *O*-GlcNAcylation and increased OGT and OGA expression in breast, lung, and colon tumor tissues compared with the respective corresponding control tissues (Gu et al., 2010; Mi et al., 2011). A similar phenomenon was evident in patients with chronic lymphocytic leukemia (Shi et al., 2010).

There is extensive crosstalk between *O*-GlcNAcylation components and those of other common PTMs, such as phosphorylation, ubiquitylation, acetylation, and methylation. The interaction of *O*-GlcNAcylation and phosphorylation components has been well studied due to the similarly modified Ser and Thr residues (Hart et al., 2011; Song et al., 2019). *O*-GlcNAcylation is found on the Ser and Thr residues on core histones H2A Thr101, H2B Ser36, and H4 Ser47 which have been identified as phosphorylation sites (Maile et al., 2004; Olson et al., 2006; Mayya et al., 2009; Sakabe et al., 2010). Several studies have shown that *O*-GlcNAcylation can prevent the degradation of target proteins by inhibiting their ubiquitination through different mechanisms. For instance, overexpression of OGA in HepG2 cells resulted in the decreased *O*-GlcNAcylation of AKT and increased phosphorylation and activity of AKT. *O*-GlcNAcylation sites in AKT have been identified as Thr308 and Ser473, key phosphorylation sites for AKT activation. These observations suggest that the *O*-GlcNAcylation of AKT competes directly with its phosphorylation (Soesanto et al., 2008; Ruan et al., 2012; Li et al., 2013; Ruan et al., 2013; Shi et al., 2015). Because OGA has both a C-terminal histone acetyltransferase-like (HAT-like) domain and an N-terminal *O*-GlcNAc hydrolase domain, *O*-GlcNAcylation and acetylation can be regulated reciprocally (Allison et al., 2012; Hayakawa et al., 2013). Other studies have shown that OGT regulates transcription, especially at transcriptional start sites, in a TET2-dependent manner, suggesting that *O*-GlcNAcylation and DNA methylation synergistically regulate gene transcription (Chen et al., 2013b; Deplus et al., 2013; Shi et al., 2013; Vella et al., 2013; Dehennaut et al., 2014; Zhang et al., 2014).

#### Citrulline and citrullination

Citrulline is a noncoding amino acid and its metabolism is categorized into two types: free citrulline from the arginine-coupled urea cycle and citrullinated proteins. Here, we focus on the second type of citrulline and discuss the mechanism and significance of citrullination (also known as arginine deimination) (Fig. 2). Histone citrulllination affects approximately 10% of all histone molecules. This less-noticed PTM converts arginine residues to citrulline by peptidyl-arginine deiminase (PAD or PADI) enzymes, resulting in reduced hydrogen bonding and a looser chromatin structure. PADs contain five Ca^2+^-dependent enzymes (PAD 1-4 and PAD6) (Witalison et al., 2015; Audia and Campbell, 2016; Yuzhalin, 2019) (Fig. 2). Citrullination modification is known to play essential roles in autoimmune and inflammatory diseases such as rheumatoid arthritis (Darrah and Andrade, 2018), periodontitis (Konig et al., 2016), autoimmune encephalomyelitis (Carrillo-Vico et al., 2010), systemic lupus erythematosus (Knight et al., 2015), and other diseases such as multiple sclerosis, atherosclerosis, thrombosis, and inflammatory bowel disease (Pritzker et al., 2000; Moscarello et al., 2007; Caprariello et al., 2018). PAD4 has been found to be a prognostic biomarker due to its increased expression in various solid tumors compared to that on the respective normal tissues (Chang et al., 2009; Chang et al., 2011) and in the peripheral blood of patients with lung cancer (Ulivi et al., 2013). In malignant lymphomas, PAD4 is also expressed in approximately 40% of cells, suggesting that its expression is associated with the development of this blood disease (Chang et al., 2009). Benign and nontumorous inflammatory tissues do not express PAD4, while metastatic tumors show higher PAD4 levels than corresponding primary tumors, suggesting that citrullination plays a role in the progression of benign tumors to aggressive malignancies (Yuzhalin et al., 2018). In colon cancer cells, knocking down PAD4 increases the expression of p53 and its target genes, leading to cell cycle arrest and cell apoptosis (Li et al., 2008). Inhibition of PADs by their inhibitor CI-amidine leads to the upregulation of OKL38 (oxidative stress-induced growth inhibitor 1), which promotes cell apoptosis and mitochondrial dysfunction in breast and osteosarcoma cells (Yao et al., 2008). Another PAD inhibitor, YW3-56, inhibits tumor growth by interfering with autophagy and regulating the Sestrin 2-mTORC1 signaling axis (Wang et al., 2012).

Among the PAD family members, PAD4 is the only protein with a nuclear localization signal (NLS) and has been reported to citrullinate histone H3 (Arg 2, 8, 17, 26), H2A, H4 (Arg 3), and H1 (Arg 54) (Wang et al., 2004; Tanikawa et al., 2012; Christophorou et al., 2014). The citrullination of H3 has been reported to open chromatin and promote gene transcription (Fuhrmann and Thompson, 2016), and citrullinated histone H3, which is mediated by PAD4, plays a vital role in the release of neutrophil extracellular traps (NETs) (Li et al., 2010). NETs have been detected in several human cancer types (Berger-Achituv et al., 2013; Merza et al., 2015; Yang et al., 2015; Tohme et al., 2016) and have been found to promote the proliferation and metastasis of cancer cells (Demers et al., 2016; Albrengues et al., 2018; Monti et al., 2018). Thålin et al. found that a 3-fold increase in citrullinated histone H3 is associated with neutrophil activation markers neutrophil elastase (NE) and myeloperoxidase (MPO) and the inflammatory cytokines IL-6 and IL-8 in 60 patients with different advanced cancers (Thalin et al., 2018). Mauracher et al. found that NET formation is associated with H3 citrullination and the occurrence of venous thromboembolism (VTE) in cancer patients (Mauracher et al., 2018). These studies suggest that citrullinated histone H3 is a novel prognostic marker for cancer detection. Upon chemotherapy treatment, PAD4 regulates cell apoptosis by interacting with p53 to citrullinate histone H4 (Arg 3) (Tanikawa et al., 2012). In addition to p53, calcium homeostasis also regulates PAD activity, and PAD-mediated citrullination is involved in calcium-mediated apoptosis (Asaga et al., 1998; Mattson and Chan, 2003; Hsu et al., 2014).

#### Itaconate and itaconation

Nearly 200 years after Swiss chemist Samuel Baup first described itaconate (itaconic acid or methylenesuccinic acid) as a product of citric acid distillation in 1836 (Luan and Medzhitov, 2016), itaconate was recently rediscovered by three groups as a microbial metabolite in the mouse lungs infected by *Mycobacterium tuberculosis* (MTB) (Shin et al., 2011), in the supernatant and cell lysates of LPS-activated RAW264.7 macrophages (Sugimoto et al., 2011) and in the intracellular compartment of glia-like VM-M3 cells (Strelko et al., 2011). Itaconate is synthesized from *cis*-aconitate in the TCA cycle of macrophages and is activated by a variety of factors, including LPS and other Toll-like receptor ligands and cytokines, such as type I and type II interferons (Shin et al., 2011; Strelko et al., 2011; Sugimoto et al., 2011; Michelucci et al., 2013). These stimuli increase the expression of aconitate decarboxylase 1 (ACOD1; also known as CAD), previously known as immune-responsive gene 1 (IRG1), which is critical for itaconate production (Lee et al., 1995; Michelucci et al., 2013). IRG1 was recently found to be induced by steroid hormone progesterone (Cheon et al., 2003), heme oxygenase-1/carbon monoxide (Uddin et al., 2016), interferon regulatory factor 1 (IRF1), the ZBP1/RIPK1/RIPK3/IRF1 axis (Tallam et al., 2016; Daniels et al., 2019) and BCAT1 (Papathanassiu et al., 2017), and suppressed by nuclear receptor Nur77 (Nr4a1) (Koenis et al., 2018) or A20 (a negative regulator of NF-κB signaling) (Van Quickelberghe et al., 2018). Itaconate, as a signal transducer, exerts anti-inflammatory effects (Murphy and O’Neill, 2018). The itaconate signal transduction mechanism is as follows: 1) it inhibits SDH and counteracts the proinflammatory signals of succinate (Cordes et al., 2016; Lampropoulou et al., 2016); 2), modifies KEAP1 to activate Nrf2 (Mills et al., 2018); and 3), and induces the ATF3-IκBζ axis, another anti-inflammatory signaling pathway (Bambouskova et al., 2018). The IRG1-itaconate-SDH axis also links innate immune tolerance with trained immunity (Dominguez-Andres et al., 2019), and IRG1 expression is important to prevent immunopathology during MTB infection (Nair et al., 2018). In glioma pathogenesis, IRG1 was identified as a novel oncogene that promotes the growth and tumorigenesis of glioma (Pan et al., 2014); IRG1-mediated itaconate production potentiates peritoneal tumor growth, and IRG1 in peritoneal tissue-resident macrophages (pResMϕ) represents a potential therapeutic target for peritoneal tumors (Weiss et al., 2018).

Recently, itaconate was found to modify KEAP1 and glutathione (Bambouskova et al., 2018; Mills et al., 2018), NLRP3 (Hooftman et al., 2020; Qin et al., 2020), ALDOA, GAPDH and LDHA (Qin et al., 2019), and other proteins involved in inflammasome, toll-like signaling, cell death, and DNA damage (Qin et al., 2020). Itaconate contains an electrophilic α,β-unsaturated carboxylic acid group, which can alkylate the cysteine residues of proteins to form a 2,3-dicarboxypropyl adduct via Michael addition. Qin et al. used a specific thiol-reactive probe, 1-OH-Az, for quantitative chemoproteomic profiling of cysteine modifications by itaconate and identified a total of 260 itaconate-modified cysteines in macrophage proteomes and showed that itaconate can modify key cysteines in glycolytic enzymes to inhibit glycolysis, which indicates its anti-inflammatory function (Qin et al., 2019; Yang, 2019). By developing a specific and cell-permeable bioorthogonal probe, itaconate-alkyne (ITalk), Qin et al. further characterized the extensive landscape of itaconate substrates (1,926 protein targets) in living cells and described this Michael addition reaction as “itaconation” (Qin et al., 2020). Moreover, itaconyl-CoA, the coenzyme A (CoA) derivative of itaconate, inhibits B12-dependent methylmalonyl-CoA mutase (MCM) by forming a stable biradical in MCM and derails its activity and repair ability (Shen et al., 2017; Ruetz et al., 2019). Although itaconate plays a decisive inflammatory role in activated macrophages and modifies a variety of proteins on cysteine residues (Luan and Medzhitov, 2016; Murphy and O’Neill, 2018; Nonnenmacher and Hiller, 2018; O’Neill and Artyomov, 2019; Yu et al., 2019), it is not clear whether itaconate affects the epigenetic remodeling of immune cells or tumor cells through itaconation, itaconyl-CoA, or other itaconate-related metabolites; hence, future studies are warranted in this regard.

## TARGETING CHROMATIN MODIFICATIONS RELATED TO METABOLISM FOR CANCER THERAPY

### Targeting acetylation for cancer therapy

Targeting the epigenetic modulation of histones or DNA to realize cancer therapy has attracted increased interest for decades. The most widely studied epigenetic inhibitors are probably HDAC inhibitors. HDAC inhibitors represent various compounds that inhibit the activity of HDACs, leading to the increased acetylation of lysine residues on histones and nonhistone proteins. The key effects of HDAC inhibitors on tumor cells are to induce cell death, cell cycle arrest, senescence, differentiation, autophagy, and tumor immunogenicity (Falkenberg and Johnstone, 2014). The short fatty acid n-butyrate was found in the mid-1970s by Riggs and colleagues to induce the differentiation of Friend erythroleukemia cells and strong histone hyperacetylation in HeLa cells (Riggs et al., 1977), and then, the groups of Allfrey and Davie reported that n-butyrate was an HDAC inhibitor (Candido et al., 1978; Vidali et al., 1978). The first US Food and Drug Administration (FDA)-approved acetylating modifier, vorinostat (also known as suberanilohydroxamic acid (SAHA)), was initially identified as a drug that induces tumor cell differentiation *in vitro* and was subsequently identified as an HDAC inhibitor (Richon et al., 1998; Mann et al., 2007). Vorinostat and another HDAC inhibitor, romidepsin, have been approved for use in cutaneous T-cell lymphoma (CTCL). Romidepsin was also approved for the treatment of peripheral T-cell lymphoma (PTCL). Their application in solid tumors has progressed slowly and remains an active research area. In addition, TSA is a commonly used HDAC inhibitor but is used only in laboratory experiments due to its high toxicity.

The pan-inhibitor nicotinamide inhibits all class III HDAC sirtuins. Sirtinol, cambinol, and EX-527 are specific SIRT1 and SIRT2 inhibitors (Ceccacci and Minucci, 2016; Eckschlager et al., 2017). They can play roles in different types of neurodegeneration and cancer (Lavu et al., 2008). In 2003, Sinclair and colleagues first found that resveratrol, a polyphenol abundant in red wine, is an activator of SIRT1 and was later named STAC (sirtuin-activating compound). STACs are chemical compounds that use NAD^+^ to remove acetyl groups from proteins. Additional results showed that resveratrol mimics caloric restriction and delays aging by extending the lifespan of metazoans and yeast (Howitz et al., 2003; Wood et al., 2004). Hubbard et al. showed that a single amino acid, Glu^230^ located in a structured N-terminal domain of SIRT1, is critical for activation of SIRT1 induced by STACs through a common allosteric mechanism (Hubbard et al., 2013). SIRT6, activated by free fatty acids such as oleic and linoleic acids, is a tumor suppressor that suppresses glycolysis (Feldman et al., 2013). The development of small-molecule activators of SIRT6 may be able to target specific tumors with low SIRT6 expression. Therefore, the field of drug development regarding SIRTs may gradually shift from inhibitors to activators, such as activating SIRT1 or SIRT6 to target age-related degenerative diseases and tumors with specific phenotypes, respectively.

In contrast to targeting writers and erasers by inhibiting their catalytic domains, effective targeting of readers needs to disrupt the protein-protein interaction. JQ-1 and I-BET are the first two compounds that can inhibit bromodomain-containing BET (bromodomain and extraterminal) proteins by binding to the bromodomain of the BET proteins (Filippakopoulos et al., 2010; Nicodeme et al., 2010; Zuber et al., 2011). JQ-1 binds the first bromodomain of the BET protein BRD4, which is a key tethering factor that interacts with cyclin T1 and cdk9 to form a core positive transcription elongation factor b (P-TEFb) (Jang et al., 2005). A more exciting effect of BET inhibition is its ability to downregulate previously undruggable *MYC* oncogene (Delmore et al., 2011; Mertz et al., 2011; Sun and Gao, 2017). JQ-1 or I-BET treatment leads to the transcriptional suppression of MYC target genes, resulting in antitumor effects in MYC-driven models of AML (Dawson et al., 2011; Zuber et al., 2011), Burkitt’s lymphoma (Mertz et al., 2011) and multiple myeloma (Delmore et al., 2011). BET inhibition also promotes immunotherapy by inhibiting the interaction of BRD2 and/or BRD4 with CDK9 and suppressing the expression of inflammatory cytokines (Nicodeme et al., 2010; Bandukwala et al., 2012).

ACLY, ACSS2 and PDC have been previously mentioned as direct effectors of acetyl-coenzyme producers in the nucleus for histone acetylation. Therefore, ACLY, ACSS2 and PDC inhibitors may now be considered new drugs for targeting the metabolic-epigenomic axis in addition to their current uses as metabolic regulators. As described above, SB-204990 is a preclinical inhibitor that specifically targets the ACLY enzyme (Pearce et al., 1998; Hatzivassiliou et al., 2005). BMS-303141 (preclinical) (Li et al., 2007), ETC-1002 (phase II clinical trial) (Ballantyne et al., 2013; Filippov et al., 2014; Gutierrez et al., 2014), and hydroxycitrate (phase IV clinical trial) (Onakpoya et al., 2011; Madeo et al., 2014) are other ACLY inhibitors in clinical trials. 1-(2,3-Di(thiophen-2-yl)quinoxalin-6-yl)-3-(2-methoxyethyl)urea is the most potent and specific inhibitor of ACSS2, which was first reported by Comerford and his colleagues (Comerford et al., 2014). Dichloroacetate (DCA), a classic PDK inhibitor, is considered a PDC activator that inhibits PDK activity. The reactivation of PDC by DCA therapy rectifies the balance between the demand and supply of oxygen, leading to cancer cell death (Michelakis et al., 2010; Dunbar et al., 2014; Chu et al., 2015; Zhang et al., 2015). Please see Table S1 for more details about the clinical trials of targeting acetylation for cancer therapy, including the inhibitors not mentioned in this review article due to space limitations.

### Targeting the methylation of DNA and histones for cancer therapy

As indicated above, the frequent hypermethylation of tumor suppressor genes further promotes cancer development; therefore, the demethylation of DNA by blockading DNMTs constitutes an interesting treatment strategy due to its reversibility. Nucleoside analogs and nonnucleoside inhibitors are the two main types of DNMT inhibitors (DNMTi), and nucleoside analogs have been known and studied for many years. Azacitidine (also known as 5-azacytidine) and decitabine (also known as 5-aza-2’-deoxycytidine) are the oldest DNMTis and are nucleoside analogs that incorporate DNA during the S-phase of the cell cycle to form an irreversible complex with DNMTs, leading to the degradation of the DNMTs. They have been approved by FDA as DNA-demethylating drugs used to treat myelodysplastic syndrome (MDS), chronic myelomonocytic leukemia, and a range of other malignancies (Kaminskas et al., 2005a; Kaminskas et al., 2005b) and represent two of the most successful and long-standing inhibitors to target epigenetic processes. Azacitidine and decitabine were first synthesized almost 60 years ago (Evans and Mengel, 1964; Sorm et al., 1964; Christman, 2002). Azacitidine has been shown to have extensive antimetabolic activity in cultured cancer cells and is an effective chemotherapeutic agent in the treatment of acute myelogenous leukemia. However, one limitation of these drugs is that they have a short half-life of approximately 30 min, which limits their exposure to diseased cells, which may thus impair their effectiveness (Marcucci et al., 2005; Karahoca and Momparler, 2013), resulting in no major responses observed in their treatment of solid tumors. Zebularine, a novel nucleoside, is a cytidine analog that is less toxic and can therefore be taken in high doses consistently (Holleran et al., 2005). Guadecitabine (SGI-110), a second-generation DNMT inhibitor, acts as a prodrug of decitabine, conferring better stability and reduced toxicity in tumor-infected nude mice compared to decitabine (Yoo et al., 2007; Chuang et al., 2010), and it has been tested in phase II clinical trials for the treatment of MDS and AML (ClinicalTrials.gov Identifier: NCT01261312).

The identification of small nonnucleoside DNMT inhibitors such as flavonoids (or bioflavonoids, such as epigallocatechin-3-gallate (EGCG)) (Galeotti et al., 2008; Yang et al., 2009; Singh et al., 2011), hydralazine (Chuang et al., 2005; Singh et al., 2009a), procainamide and procaine (Yoo and Medina-Franco, 2011), curcumin (Liu et al., 2009; Shu et al., 2011), RG108 (phthalimido-L-tryptophan) (Siedlecki et al., 2003; Brueckner et al., 2005), SGI-1027 (Denny et al., 1979; Datta et al., 2009), and MG98 (Goffin and Eisenhauer, 2002; Amato, 2007), which bind directly to DNMT catalytic regions without binding to DNA, has also attracted considerable attention. However, *in vitro* studies have shown that nonnucleoside compounds induce limited epigenetic changes in living cells (Ren et al., 2011), and none of the afore mentioned nonnucleoside DNMT inhibitors has entered clinical development; thus, there is still a long way to go before novel, selective, nonnucleoside DNMT inhibitors will be available for clinical research.

There are fewer effective inhibitors of HMTs and KDMs than inhibitors of acetylation and DNA methylation. Over the past few years, the number of histone methylation small-molecule modulators has increased rapidly through significantly increased efforts in academia and the pharmaceutical industry (Liu et al., 2014; Liu et al., 2015; McGrath and Trojer, 2015). Inhibitors of the histone methyltransferases DOT1L (Yu et al., 2012; Daigle et al., 2013) and EZH2 (Knutson et al., 2012; McCabe et al., 2012) have exciting potential for cancer treatment.

*DOT1L* (disruptor of telomeric silencing 1-like) was identified as a human homolog of *Dot1*, a gene found in the yeast *Saccharomyces cerevisiae* (Singer et al., 1998). DOT1L catalyzes histone H3 lysine 79 (H3K79) methylation by transferring a methyl group from its substrate SAM to the amino group of lysine residues. It is the only enzyme critical for the monomethylation, dimethylation, and trimethylation of the ε-amino group on H3K79 (Feng et al., 2002; Lacoste et al., 2002). EPZ004777, the first SAM-competitive inhibitor of DOT1L, was able to kill biphenotypic (mixed-lineage) leukemia (MLL)-rearranged leukemia cells and prolong the survival time of mice with *MLL*-rearranged leukemia, but it had little killing effect on non-MLL translocated cells (Daigle et al., 2011; Chen et al., 2013a). By occupying the SAM-binding pocket and inducing conformational changes in DOT1L, EPZ-5676, a derivative of EPZ004777, is another effective DOT1L inhibitor that led to tumor regression in rat xenograft models of *MLL*-rearranged leukemia (Daigle et al., 2013).

H3K27 methyltransferase EZH2 is the catalytic component of PRC2, an established transcriptional repressor (Cao et al., 2002; Levine et al., 2004). Several studies have shown that EZH2 disorders are often associated with the progression, metastasis, and poor clinical outcomes of patients with lymphoma and solid tumors, including prostate, breast, kidney, and lung cancers (Varambally et al., 2002; Moss and Wallrath, 2007; Yu et al., 2007; Varambally et al., 2008; Wagener et al., 2010; Takawa et al., 2011; McCabe et al., 2012; Volkel et al., 2015). GSK126 effectively inhibits wild-type and mutant EZH2 methyltransferase activity by competing with SAM. GSK126 can inhibit the proliferation of EZH2-mutant diffuse large B-cell lymphoma (DLBCL) cells and markedly inhibit the growth of EZH2-mutant DLBCL xenografts in mice (McCabe et al., 2012). Subsequently, other EZH2 inhibitors, including EPZ005687 (Knutson et al., 2012), EPZ-6438 (tazemetostat) (Knutson et al., 2014), and CPI-1205 (Taplin et al., 2018), have been developed and are currently in phase I or II clinical trials.

IDH mutations indirectly inhibit extensive histone demethylases and lead to histone hypermethylation throughout the genome. Targeting IDH mutations for glioma and AML appears to be a promising therapeutic approach. The IDH1^R132H^ inhibitor (AGI-5198) impairs the growth and promotes the differentiation of glioma cells with the IDH1^R132H^ mutation by inducing the demethylation of histone H3K9me3 and the expression of genes critical for gliogenic differentiation (Rohle et al., 2013). AGI-6780, a potent and selective inhibitor of the tumor-associated mutant IDH2^R140Q^, binds IDH2 at the dimer interface. Treatment of TF-1 human erythroleukemia cells and primary human AML cells with AGI-6780 induces cellular differentiation (Wang et al., 2013). AG-221, another selective inhibitor of the mutant IDH2^R140Q^ enzyme, suppresses 2HG production and induces the differentiation of both human AML cells and mouse model cells (Yen et al., 2017). These inhibitors provide potential applications as differentiation therapies for cancer, at least for AML. In addition, the frequent deletion of methylthioadenosine phosphorylase (MTAP) as a consequence of 9p21 loss in cancer cells leads to the dysregulation of methionine metabolism and makes tumor cells more sensitive to protein arginine *N*-methyltransferase 5 (PRMT5) inhibitors, creating a new therapeutic opportunity based on methionine metabolism and epigenomic interactions (Kryukov et al., 2016; Marjon et al., 2016; Mavrakis et al., 2016).

DNA methylation and histone acetylation are the earliest epigenetic targets for drug development. As described above, epigenetic drugs, including DNMT and HDAC inhibitors, have been approved by FDA for clinical use with hematologic malignancies and other cancers. In contrast, there is still considerable room for further development of targeted histone methylation in terms of mechanistic discovery and drug intervention. Moreover, the success of FDA-approved drugs for use with solid tumors has been limited because of the specific tumor microenvironment of these cancers, such as hypoxia and immune cells. Nevertheless, to date, there are many epigenetic targets of proteins and DNA that have not been properly assessed with inhibitors; therefore, epigenetic therapies, along with other therapies, may offer many opportunities for tumor therapy. For more information about the usage of HDAC inhibitors, IDH1/2 inhibitors, SAM cycle inhibitors and other inhibitors or activators of epigenetic modulation in cancer, please see the excellent reviews by Falkenberg et al., Eckschlager et al., Wong et al. and Cheng et al. (Falkenberg and Johnstone, 2014; Eckschlager et al., 2017; Wong et al., 2017; Cheng et al., 2019). Please see Table S2 for more details about the clinical trials of targeting methylation for cancer therapy, including the inhibitors not mentioned in this review article due to space limitations.

### Dietary interventions that target epigenetically modified proteins and metabolic molecules as cancer therapy

**Figure 4:**
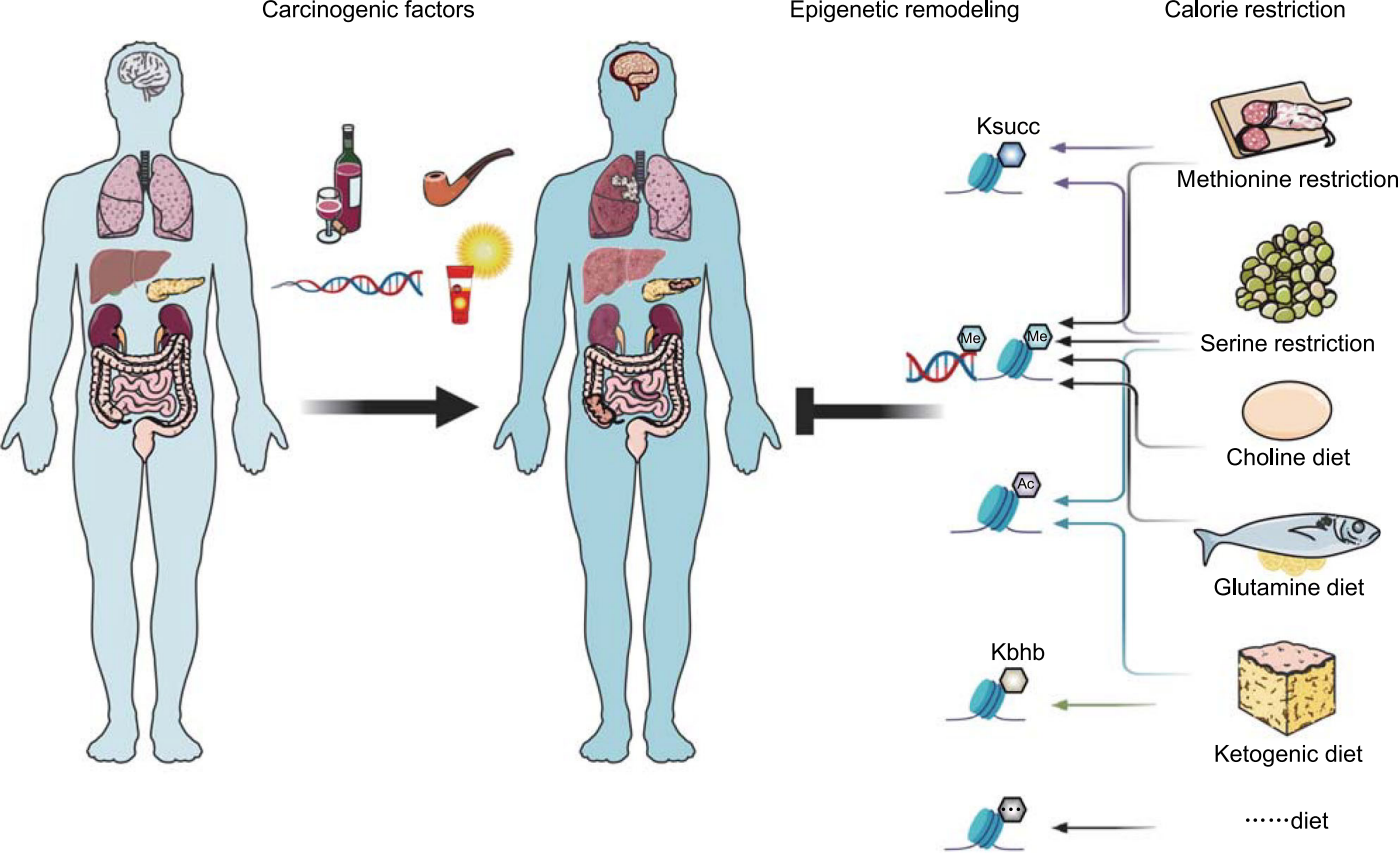
**Dietary-based approaches for cancer therapy**. Genetic and environmental factors, including gene mutation, radiation, smoking, and excessive drinking, can cause a variety of human diseases, such as glioma, liver cancer, lung cancer, pancreatic cancer, kidney cancer, and colorectal cancer, which are associated with metabolic dysregulation and epigenetic remodeling. Dietary intake regulates nutrient availability, metabolite generation, and epigenetic modifications. Dietary changes in the composition of ketones (low carb, high fat diet, such as yogurt, eggs), glutamine (dietary fish, soybean), choline (dietary eggs, meat, fish), methionine (methionine restriction: dietary less proteins, it is only tested on animals), or serine (serine restriction: serine- and glycine-free diet, it is only tested on animals), may extend lifespan and have health-promoting effects by reshaping the homeostasis of metabolism and epigenetics such as methylation, acetylation, succinylation, and β-hydroxybutyrylation

Calorie restriction and fasting have been shown to extend lifespan and confer health-promoting effects. Recently, an increasing number of studies have discovered that the disruption of the dietary components of methionine (Xu et al., 2020), serine and glycine (Maddocks et al., 2017; Muthusamy et al., 2020), ketone bodies (Xia et al., 2017; Hopkins et al., 2018), choline (Romano et al., 2017), arginine (Poillet-Perez et al., 2018), glutamine (Ishak Gabra et al., 2020), fructose (Goncalves et al., 2019; Zhao et al., 2020), or cysteine (Badgley et al., 2020) mediates the progression of multiple types of cancer. Here, we review the latest progress in dietary controls in cancer therapy at the epigenetic level (Fig. 4).

Ketogenic diets are high-fat low-carbohydrate diets that inhibit cancer progression, in contrast to high-fat, high-carbohydrate diets that induce obesity and promote cancer progression (Branco et al., 2016). A ketogenic diet promotes the β-oxidation of fatty acids and the generation of ketone bodies, such as AcAc, β-OHB, and acetone, in the livers. Ketogenic diets function as “insulin-suppressing diets” by reducing circulating insulin and insulin-like growth factor-1 (IGF-1) levels, thereby limiting the aberrant activation of oncogenes (Nencioni et al., 2018; Klement, 2019). Insulin feedback induced by PI3K inhibitor treatment reactivates the PI3K-mTOR signaling axis in tumors, which may significantly negate the beneficial therapeutic effects of inhibitors. Feeding ketogenic diets together with a PI3K inhibitor to decrease hyperglycemia and lower insulin release resulted in the improved survival of the mice bearing PI3K-driven tumors (Hopkins et al., 2018). However, ketogenic diets alone had varying effects on different tumor models. For example, accelerated disease progression was shown for AML mouse models fed ketogenic diets alone (Hopkins et al., 2018), and these diets also accelerated tumor growth in other rodent tumor models (Liskiewicz et al., 2016; Xia et al., 2017). Whether ketone body-derived acetyl-CoA and β-hydroxybutyrate and the subsequent acetylation and β-hydroxybutyrylation are involved in the confusing effect of ketogenic diets on different tumor growth remains unclear.

Although glutamine is the most abundant amino acid in the culture medium and is essential for immune cell function and tumor development (Gao et al., 2009; Altman et al., 2016; Kelly and Pearce, 2020), Ishak Gabra et al. found that glutamine supplementation in the diet suppresses melanoma tumor growth independent of BRAF status. Dietary glutamine-derived α-KG levels *in vivo* led to the hypomethylation of H3K4me3, thereby inhibiting epigenetically activated oncogenic pathways in melanoma (Ishak Gabra et al., 2020). This study showed the potential of dietary intervention with glutamine to block melanoma tumor growth by epigenetic reprogramming. Choline is an essential methyl donor in one-carbon metabolism for the methylation of histones and DNA. Romano et al. found that choline-utilizing bacteria compete with the host to consume choline, affecting the plasma and hepatic levels of methyl donor metabolites in the host. Mice with choline-consuming bacteria exhibit increased susceptibility to metabolic disease upon depletion of methyl donor metabolites when fed a high-fat diet (Romano et al., 2017). Excessive consumption of fructose increases the incidence of obesity and nonalcoholic fatty liver disease (Hannou et al., 2018; Jensen et al., 2018). *In vivo* isotope tracing revealed that the gut microbiota decomposes dietary fructose into acetate, providing acetyl-CoA for lipogenesis and H3K27 acetylation within the *ACSS2* genomic locus. Depletion of the microbiota or hepatic ACSS2 suppresses acetyl-CoA generation and the synthesis of fatty acids (Zhao et al., 2020).

Dietary restriction of methionine as a therapeutic approach was proposed over 60 years ago (Sugimura et al., 1959). A methionine-limited diet suppresses tumor invasion and metastasis (Breillout et al., 1987; Guo et al., 1996; Jeon et al., 2016). The combination of a methionine-limited diet and one-carbon metabolism inhibitors, such as 5-fluorouracil (5-FU), has a synergistic effect on tumor inhibition (Hoshiya et al., 1997; Xiao et al., 2001). Methionine supplementation in tumors restores T-cell function in B16F10 tumor-bearing mice and retards tumor growth (Bian et al., 2020). However, notably, methionine restriction together with choline depletion promotes hepatic injury in some rodent models (Caballero et al., 2010). Dietary restriction of serine and glycine decelerates tumor growth and increases mouse survival (Maddocks et al., 2013; Gravel et al., 2014; Maddocks et al., 2017; Muthusamy et al., 2020). However, it is not clear whether the antitumor effect of serine and glycine starvation depends on the alteration of methylation status. Please see Table S3 for more details about the clinical trials of targeting metabolic enzymes for cancer therapy, including the inhibitors not mentioned in this review article due to space limitations. Moreover, Table S4 summarizes the information of the clinical trials in combination with epigenetic drugs and metabolism-targeting drugs for cancer therapy in recent years.

## CONCLUSIONS AND FUTURE PERSPECTIVES

Substantial progress has been made in the understanding and research on the intersection of tumor metabolism and epigenetics in recent decades. However, there are still many important scientific questions to be answered. For example, what are the specific action and mechanism of nonacetyl histone acylation modulations, including propionylation, butyrylation, crotonylation, malonylation, 2-hydroxyisobutyrylation and glutarylation, in the development of different cancers (Sabari et al., 2017; Zhao et al., 2018). When multiple modification pathways target the same amino acid residues, there may be competitive antagonism between different modifications. This is especially true for the ε group of lysine, which can be acetylated, methylated, or ubiquitinated (Bannister and Kouzarides, 2011; Sadakierska-Chudy and Filip, 2015; Zheng et al., 2020), such as the balance between H3K9 acetylation and methylation (Nicolas et al., 2003), dynamic competition of H4 K5K8 acetylation and butyrylation (Goudarzi et al., 2016), and the overlap of succinylation sites and acetylation sites in diverse organisms including bacteria, yeast, and human cells (Weinert et al., 2013). Itaconate, produced mainly by stimulated macrophages, can modify protein alkylation and protein itaconation (Lampropoulou et al., 2016; Bambouskova et al., 2018; Mills et al., 2018; Qin et al., 2019; Qin et al., 2020), but it is unclear whether it directly modifies histones or indirectly modify histones through succinate to regulate gene expression and cancer progression. Another largely unanswered question is, are certain regions of chromatin more susceptible to metabolic rewiring than other regions.

Localized metabolites, especially in the nucleus and mitochondria, reveal the importance of enzyme translocation in the regulation of epigenetics. Thus, discovering the roles of metabolites in other organelles, such as lysosomes, the endoplasmic reticulum, Golgi apparatus, and storage granules, will be crucial to understanding how metabolism and epigenetics interact with each other. Of particular importance, α-KGDH localized in the nucleus serves as a succinyltransferase to succinylate histones (Wang et al., 2017b; Xu et al., 2021), and CPT1A can also serve as a lysine succinyltransferase upon the succinylation of S100A10 (Kurmi et al., 2018; Wang et al., 2019a). Other aforementioned nuclear localized metabolism enzymes, such as PKM2, ACLY, and PDC, not only provide the corresponding substrates or metabolites for chromatin modulation but also interact with other proteins to form complexes. Detailed analyses of the interacting partners and elucidation of their nonmetabolic or moonlighting roles are underappreciated opportunities but important for us to understand and battle cancers.

Many clinical trials are testing epigenetic molecular inhibitors, such as HDAC inhibitors and DNMT inhibitors. The IDH1-mutant inhibitor ivosidenib and IDH2-mutant inhibitor enasidenib are granted by FDA for the treatment of AML with IDH1 or IDH2 mutations, respectively (Table S3). Dietary therapy is another promising antitumor approach that seems to be more convenient and economical and, despite desirable outcomes in animal studies, requires significant research before reaching the clinical stage.

The metabolic rewiring of tumor and immune cells regulates tumor progression by shaping the epigenome in the tumor microenvironment. While several typical PTMs modulated by metabolic enzymes and metabolites are reviewed here, the metabolome-epigenome crosstalk paradigm is continuously expanding, and the elucidation of the molecular basis of these PTMs will provide us with exciting opportunities to efficiently combat cancer.

## Electronic supplementary material

Below is the link to the electronic supplementary material.

Electronic supplementary material 1 (XLSX 50 kb)
